# Probing Ultrastrong Through‐Space Electronic Coupling in Donor‐Acceptor Systems at the Single‐Molecule Level

**DOI:** 10.1002/advs.202521879

**Published:** 2026-01-04

**Authors:** Xin Wang, Dan Yang, Jiazheng Diao, Jens Ulstrup, Chengyang Zhang, Florian Auras, Qiang Fu, Jinlong Yang, Linsong Cui, Yueqi Li, Jinghong Li

**Affiliations:** ^1^ Hefei National Research Center For Physical Sciences At the Microscale University of Science and Technology of China Hefei China; ^2^ Key Laboratory of Precision and Intelligent Chemistry University of Science and Technology of China Hefei China; ^3^ Center for Bioanalytical Chemistry University of Science and Technology of China Hefei China; ^4^ Department of Chemistry Technical University of Denmark Kongens Lyngby Denmark; ^5^ Faculty of Chemistry and Food Chemistry TUD Dresden University of Technology Dresden Germany; ^6^ Department of Chemistry Key Lab of Bioorganic Phosphorus Chemistry and Chemical Biology Tsinghua University Beijing China

**Keywords:** donor‐acceptor systems, electron transfer, scanning tunneling microscopy‐break junction, single‐molecule conductance, through‐space interactions

## Abstract

Through‐space donor‐acceptor (TSDA) interactions have recently emerged as a new paradigm for charge transfer and transport in organic semiconductors. However, the intrinsic D–A coupling strength and charge transport properties at the molecular level remain unexplored in comparison to established through‐bond channels. Here, we employ a variety of single‐molecule techniques to directly probe TSDA charge transport characteristics in a series of strategically designed face‐to‐face D–A systems, revealing that optimized spatial proximity and electronic complementarity yield conductance values up to ∼0.19 G_0_, comparable to the best‐performing through‐bond molecular wires. Flicker noise analysis, current‐voltage characterization, and mechanical stretching measurements confirm robust electronic and mechanical coupling, while theoretical calculations and photophysical studies identify through‐space transmission as the prevailing mechanism. These findings establish non‐covalent TSDA coupling as an efficient charge‐transport channel and provide new molecular‐level insight for the design of D–A systems in organic electronics and optoelectronics.

## Graphical abstract

A series of through‐space coupled donor‐acceptor (D–A) molecules is sandwiched between two electrodes to determine their single‐molecule conductance. As the D–A interaction weakens, single‐molecule conductance reduces while Flicker Noise PSD n‐value increases (coupling strength decreases). Especially for TS1, the conductance reaches 0.19 G_0_, and its ultra‐strong electronic coupling behaves like through‐bond interactions. Such results are entirely novel in single‐molecule electronics.

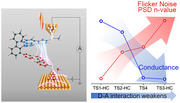

## 1 Introduction

Charge transfer between molecular donor (D) and acceptor (A) units is a central mechanism in the electronic function of a wide range of organic materials, from light‐emitting diodes [1, 2] and solar cells [3, 4, 5] to molecular‐scale electronic devices [6, 7, 8, 9]. While such interactions are typically mediated through covalent bonds (through‐bond coupling) [10], recent attention has turned toward through‐space charge transfer (TSCT), where D and A units interact without direct covalent bonding, often via *π–π* stacking [11, 12, 13]. Despite growing interest, the nature and strength of through‐space donor‐acceptor (TSDA) interactions at the single‐molecule level remain poorly understood. In particular, it is unclear whether spatially aligned D–A pairs can support electronic coupling strong enough to rival that of through‐bond systems, and how these interactions depend on distance, orientation, and electronic character. Addressing these fundamental questions requires direct, single‐molecule studies beyond ensemble‐averaged optoelectronic studies of bulk or thin‐film materials [14, 15].

Here, we address this gap by a variety of single‐molecule electronic measurements [16, 17, 18, 19, 20, 21] to directly probe TSDA interactions in a series of strategically chosen D‐bridge‐A molecules comprising dihydroacridine donors, borane acceptors, and a non‐conjugated fluorene bridge. By constraining donor and acceptor moieties in a face‐to‐face geometry and systematically tuning the acceptor electronic properties, we elucidate how spatial proximity and electronic complementarity govern charge transport. Using core single‐molecule techniques, including conductance measurements [16], flicker noise analysis [19], current‐voltage (*I–V*) characterization [22], and mechanical stretching analysis [23, 24], we qualitatively and quantitatively characterize through‐space interactions at the single‐molecule level. Previous single‐molecule studies have suggested that intramolecular through‐space interactions can enhance molecular conductance [25, 26]. Although the enhancement was relatively modest and only a limited set of techniques was applied, these studies still demonstrate the power of electronic measurements for probing through‐space coupling.

Our combined experimental and theoretical study shows that strong TSDA coupling can yield remarkably high single‐molecule conductance. The molecule TS1, featuring a strongly electron‐deficient acceptor and optimal D–A alignment, exhibits a conductance of ∼0.19 G_0_, surpassing many state‐of‐the‐art through‐bond molecular wires [27, 28]. Flicker noise power, *I–V* characteristics, and junction stretching analyses reveal robust electronic and mechanical coupling between the D and A units, comparable to those of established through‐bond systems [19], while density functional theory and non‐equilibrium Green's function transport calculations confirm through‐space transmission as the prevailing mechanism. Photophysical measurements further disclose distinct charge‐transfer absorption and intense luminescence, consistent with strong electronic coupling between the donor and acceptor moieties. Together, these findings establish that non‐covalent TSDA interactions can mediate ultra‐strong charge transport on par with covalent pathways, offering new molecular‐level design principles for donor–acceptor systems in thermally activated delayed fluorescence (TADF) and molecular electronics.

## 2 Results and Discussion

### 2.1 Molecular Design and Photophysical Properties

**FIGURE 1:**
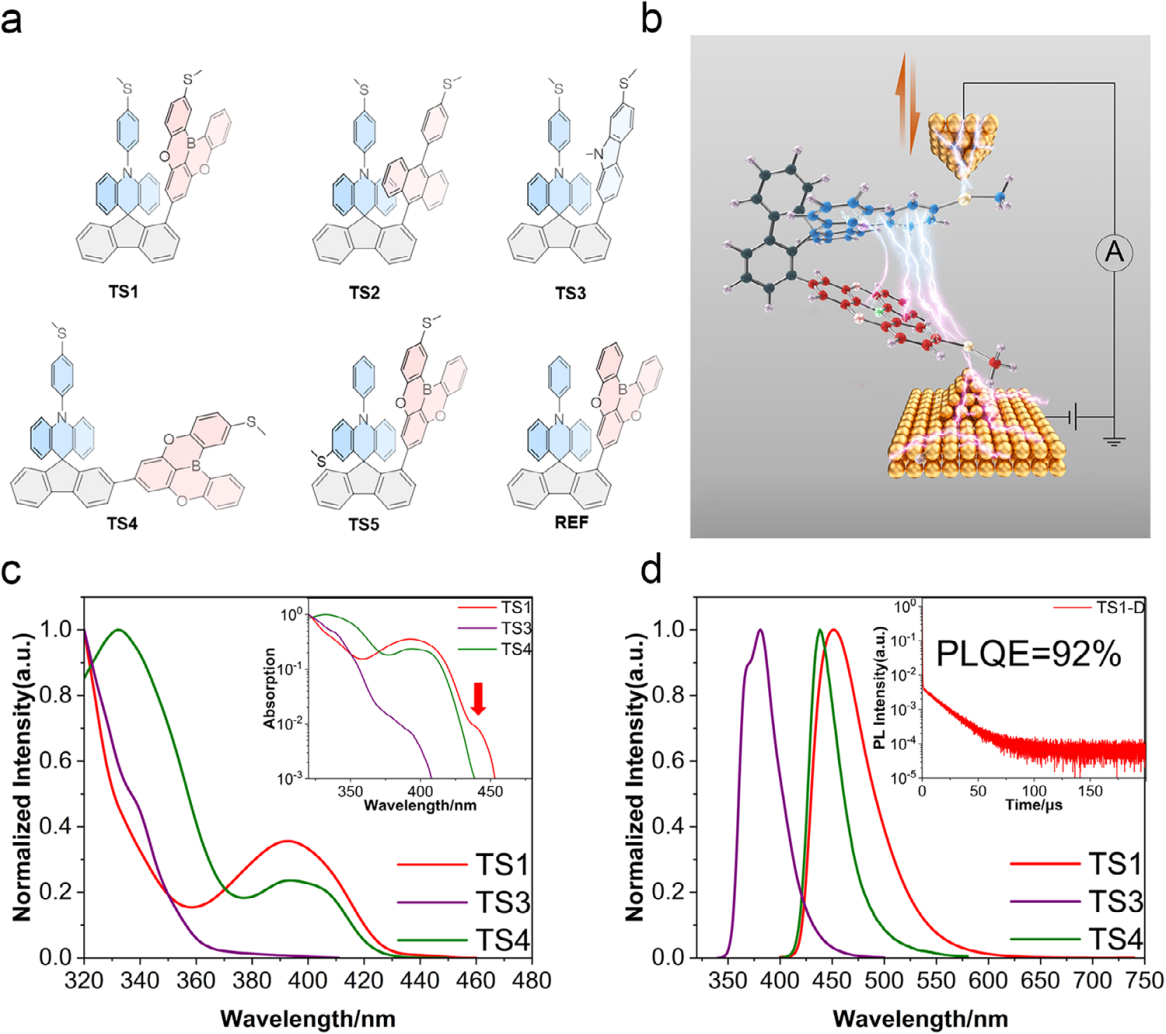
Molecular structures, UV–vis absorption and fluorescence spectra of the charge‐transfer molecules, and a schematic illustration of the single‐molecule conductance recordings. (a) Chemical structures of TS1‐TS5 and REF. The electron‐rich donor moieties are shown in blue and the electron‐deficient acceptor units in red. (b) Schematic illustration of the scanning tunneling microscopy break junction (STM‐BJ) measurement for a TS1 molecule with a strong through‐space donor‐acceptor interaction. The up and down arrows indicate the tip movement during the junction formation and breaking process. The atoms of the electron‐rich donor moieties are shown in blue, and those of the electron‐deficient acceptor units in red. (c) Normalized absorption spectra (room temperature, 1 × 10^−5^ m in mesitylene) of TS1, TS3, and TS4; the inset is the absorption with a log coordinate. The feature at 440 nm reflects the D–A charge transfer transition. (d) Normalized fluorescence (room temperature, 1 × 10^−5^ m in mesitylene) of TS1, TS3, and TS4; the inset is the transient PL decay of TS1 in oxygen‐free toluene solution (1 × 10^−5^ m).

In order to develop a strategy for how TSCT controls the single‐molecule charge transport between D and A, we designed and prepared a series of molecules with different strategically chosen geometries and electronic configurations (Figure 1a). As a proof‐of‐concept, we employed 10‐phenyl‐9,10‐dihydroacridine as the donor unit, fused to a rigid fluorene bridge. A triphenylborane‐derived acceptor unit was incorporated either at the 1‐position of the fluorene bridge (TS1, TS5, and REF) to achieve a closely coupled D–A configuration, or at the 2‐position (TS4) to increase the D–A through‐space distance, Figure 1a. In addition, TS2 and TS3 incorporate more electron‐rich anthracene or carbazole units, respectively. ─SMe groups are attached to the TS1–TS5 molecules to enable reliable Au─S bonding as anchoring points to the enclosing gold electrodes of scanning tunnelling microscopy (STM) (Figure 1b). The molecular structures are comprehensively supported by NMR spectroscopy (Figures S1–S12).

The UV–vis absorption and photoluminescence (PL) spectra of the designed TSCT molecules were recorded at room temperature. As shown in Figure 1c, the UV–vis absorption spectra of TS1, with a face‐to‐face D/A orientation, in addition to absorption bands of π–π* transitions, exhibit an additional charge transfer transition band at 440 nm, reflecting significant through‐space electronic couplings between the donor and acceptor moieties in their ground‐state geometry. In contrast, this charge transfer band is absent in the spectra of both TS4, which has an increased D/A distance and nonparallel orientation, and of TS3, which has a weak (less electron‐deficient) acceptor. TS1, TS2, and TS4 exhibit distinct emission peaks at 451, 475 , and 438 nm, respectively (Figure 1d; Figure S13). The photoluminescence quantum efficiencies (PLQEs) of the TSCT‐based molecules further exhibit marked differences. TS1 in toluene solution displays the highest PLQEs, reaching 92 %. The electronically more weakly coupled TS3 exhibits the lowest PLQE within this series, of only 11 % in toluene solution (Table S1). To better understand the emission mechanism, transient photoluminescence (PL) spectra were recorded for TS1 in degassed solutions at room temperature. TS1 exhibits a prompt 67 ns component and a distinct delayed component with a lifetime of 15.6 µs (Figure 1d, inset), indicative of efficient spin‐flip from a triplet to a singlet excited state. In contrast, TS2, TS3, and TS4 only display suppressed delayed emission, indicative of a slower and less efficient spin‐flip process (Figure S14).

### 2.2 Single‐Molecule Conductance Depends Crucially on the D–A Interaction Strength

**FIGURE 2:**
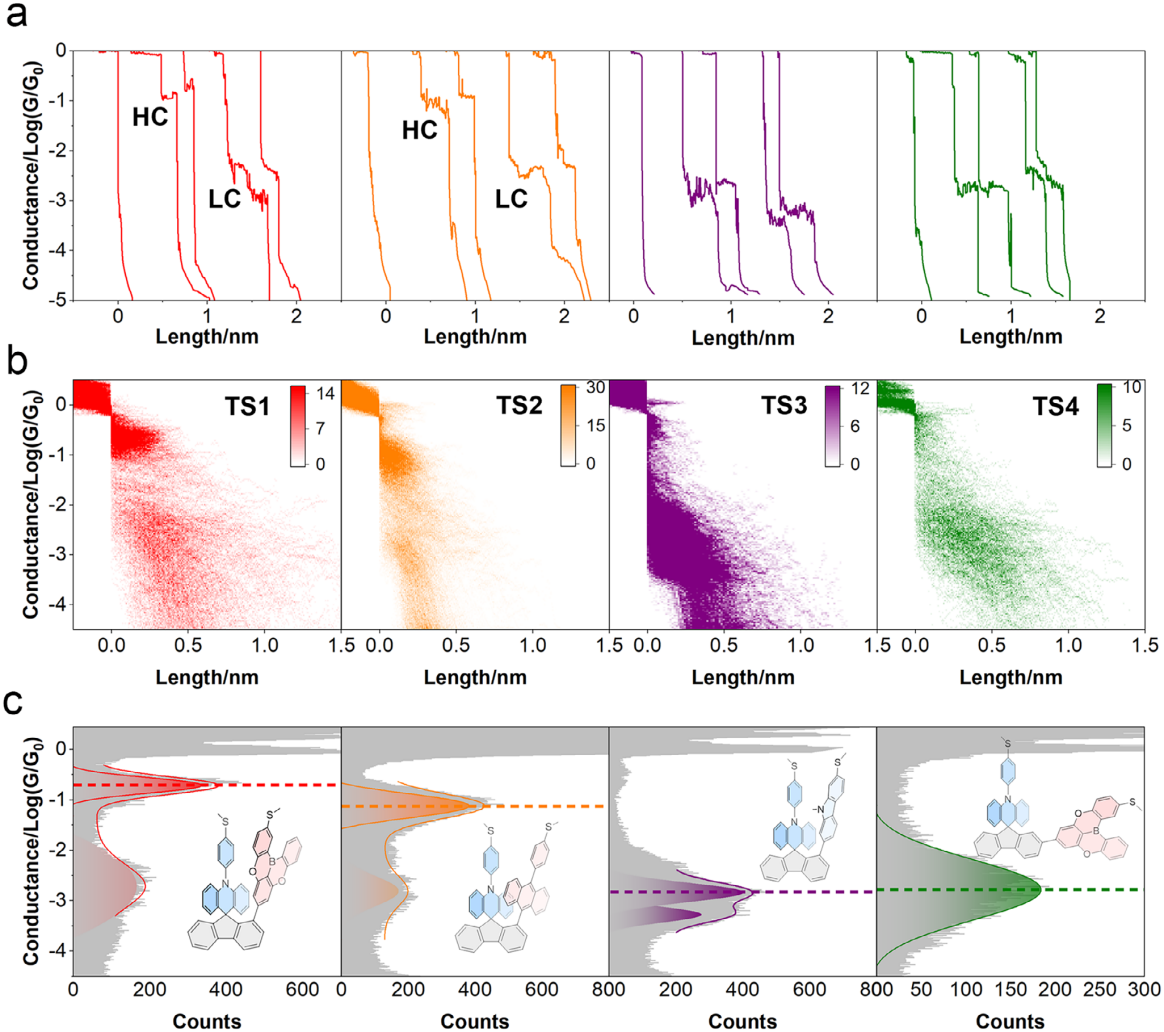
Conductance measurements of compounds TS1‐TS4. (a) Representative individual traces of conductance vs. distance of each molecule. (b) 2D conductance histograms of conductance vs. distance. Color bars indicate the number of counts at each bin. (c) 1D conductance histograms.

In order to understand in detail the nature and strength of through‐space donor‐acceptor (TSDA) interactions at the single‐molecule level, we conducted single‐molecule conductance measurements using the in situ electrochemical scanning tunnelling microscopy break‐junction (STM‐BJ) technique [16], on 100 µM mesitylene solutions of the respective compounds (Figure 2). The STM tip was repeatedly brought into and retracted from contact with the substrate probe molecule, allowing molecular junctions to form and break (see Methods for details). Individual conductance‐distance traces were obtained by recording the conductance as a function of tip displacement during retraction (Figure 2a). The appearance of a conductance plateau indicates the formation of a molecular junction, while a sharp conductance drop following the plateau marks its rupture. Control measurements of the pure solvent exhibited no discernible features (Figures S15 and S16). TS1 and TS2 displayed plateaus at two distinct conductance levels, denoted as high conductance (HC) and low conductance (LC) states. These reflect two different types of junctions. In contrast, TS3 and TS4 exhibited plateaus centred around a single level.

2D conductance histograms were constructed from hundreds of individual conductance‐distance curves that display these plateau features (see Supporting Information for automatic selection criteria and junction formation yields). Two high‐count regions were observed for TS1 and TS2, while only a single high‐count region was evident for TS3 and TS4 (Figure 2b). Closer examination of the 2D histograms shows that the plateaus of the HC states of TS1 and TS2 were shorter and less sloped compared to their LC states (Figure S17). The conductance states of TS3 and TS4 exhibited slopes similar to the LC states of TS1 and TS2. A small plateau slope suggests a stable molecule‐electrode connection via a chemical bond, while a larger slope indicates unstable coupling [29, 30], presumably via Au‐π interaction [31] in this molecular system.

1D conductance histograms constructed from the same dataset further highlight the differences between the compounds. TS1 and TS2 show two well‐separated peaks corresponding to high‐ and low‐conductance states. TS3 exhibits two tightly overlapped peaks, while TS4 displays only a single peak (Figure 2c). The conductivities of the HC and LC states are 10^−0.71^G_0_ and 10^−2.73^G_0_ for TS1 and 10^−1.11^G_0_ and 10^−2.81^G_0_ for TS2, respectively. Notably, the conductance of the HC states of TS1 and TS2 is extraordinarily high for molecules of this size, suggesting that the charge transport is not through the long donor‐bridge‐acceptor pathway via the molecular backbone [32, 33]. Conductance measurements of 1,2‐biphenyldithiol, 1,3‐biphenyldithiol and 1,4‐biphenyldithiol showed conductance values at 10^−3.04^ G_0_, 10^−2.33^ G_0_ and 10^−1.35^ G_0_ (Figure S15), respectively. Despite comparable sizes of the bridge part, these control molecules thus exhibited lower conductance than the HC states of TS1 and TS2, further supporting that charge transport is not via the donor‐bridge‐acceptor pathway. Considering the small slope of the HC state plateaus, we infer that charge transport of TS1 (HC) and TS2 (HC) is mainly via the Au─S bonds and further via the through‐space coupling between the donor and acceptor subunits. The 1D histograms for TS3 show two tightly overlapped peaks (10^−2.83^ G_0_ and 10^−3.28^ G_0_) and a wide peak for TS4 (10^−2.78^ G_0_). The peak overlapping inTS3 and the broad peak width of TS4, and in the 1D histograms, suggest mixing of two different junction geometries. Notably, all these conductance values were lower than those of the HC states of TS1 and TS2.

Comparing the conductance of the HC states discloses further the trend G_TS1(HC)_ > G_TS2(HC)_ > G_TS3(HC)_, consistent with the trend of the charge deficiency of the acceptor moiety, i.e., the more pronounced the charge deficiency, the higher the conductance. The comparison between the HC state of TS1 and the state of TS4 indicates that spatially separating the donor and acceptor leads to a substantial decrease in conductance. The conductance pattern thus demonstrates that non‐covalent D–A interaction plays a key role in the single‐molecule junction charge transport, and that the interaction is very strong for TS1 and TS2.

Conductance measurements of TS5 and REF were carried out as controls to investigate the different molecule‐electrode anchoring and binding sites. The conductance features of TS5 and REF are depicted in Figure S15. The ─SMe anchor in TS5 is located at a different site on the donor group compared to TS1, yielding a shorter through‐bridge charge transport pathway (Au‐S‐D‐B‐A‐S‐Au) and a longer through‐space pathway (Au‐S‐D‐space‐A‐S‐Au), due to the increased distance along the donor moiety (Figure S18). Notably, TS5 exhibits a single conductance peak centered at 10^−2.10^G_0_. This is lower than for G_TS1(HC)_. This behavior indicates that elongation of the through‐space pathway has a stronger impact on charge transport than shortening of the through‐bridge pathway, thereby supporting that the through‐space channel plays a dominant role in TS1. It is noteworthy that REF has no ‐SMe anchors and can only bind to the electrodes via Au‐π interactions [31]. REF displays two conductance peaks, at 10^−1.71^G_0_ and 10^−2.67^G_0_, respectively. Although G_REF(LC)_ is comparable to G_TS1(LC)_, G_REF(HC)_ is significantly lower than G_TS1(HC)_, emphasizing the critical role of covalent binding to the electrodes.

### 2.3 Flicker Noise Analysis Supports Strong D–A Coupling

To further evaluate the electronic coupling strength along the through‐space charge transport pathways, flicker noise analysis on the single molecules was conducted. Flicker noise (1/f noise) arises from the interaction of the structural fluctuations of electrode atoms with the probe molecule, the power reflecting the coupling along the charge transport pathway of the molecular junction [19, 30, 34]. When fitted to the n‐th power of the junction conductance, the power spectral density (PSD) of flicker noise reflects the strength of the electronic coupling. Typically, n values close to unity denote strong coupling, while values close to 2 suggest weaker coupling. Specifically, n∼1.2 corresponds to through‐bond charge transport, while n∼2 corresponds to through‐space charge transport [19].

**FIGURE 3:**
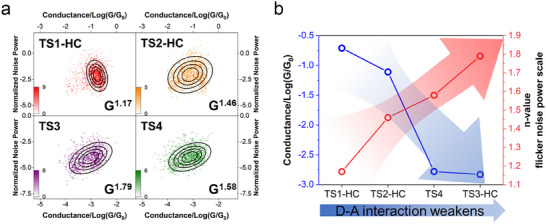
Flicker Noise analysis of compounds TS1‐TS4. (a) 2D histograms of normalized flicker noise power ((Noise power)/G) vs. average conductance. The black ellipses represent contour lines of a fitted 2D Gaussian distribution at 20 %, 40 %, 60 %, and 80 % of the Gaussian peak height. (b) Diagram of conductance and n‐value varying with the D–A interactions. This diagram shows an inverse relationship between conductance and flicker noise power across the molecular series.

In our experiments, tip movement was stopped upon detecting the formation of a single‐molecule junction, and the tunnelling current was recorded for 0.1 s for flicker noise analysis within the 100–1000 Hz frequency range. Figure 3a illustrates the PSD vs. conductance states of TS1 (HC), TS2 (HC), TS3, and TS4 (see Methods for analytical details). The PSDs of TS1 (HC) and TS2 (HC) were scaled with G^1.17^ and G^1.46^, respectively. These n values are comparable to the typical n value for through‐bond coupling (∼1.2) and substantially lower than those associated with *π–π* stacking (∼1.7) [35], σ‐σ stacking [21], and Au‐π coupling (∼1.8) [19, 36]. Given that our conductance measurement results exclude charge‐transport pathways through the non‐conjugated molecular backbone, the low n values and high conductance suggest that the strength of D–A through‐space coupling for the HC state of TS1 and TS2 falls in the same regime as coupling via chemical bonds. The results also suggest molecular binding to the electrodes via an Au─S bond.

In contrast, the n values are significantly larger for TS3 (1.79) and TS4 (1.58), indicating much weaker D–A interactions compared to TS1 (HC) and TS2 (HC). Meanwhile, the n values of the LC states of TS1 (1.75) and TS2 (1.75) and the HC (1.8) and LC (1.85) states of REF are all close to 2 (Figure S19), in agreement with reported results for Au‐π coupling [19, 36]. We therefore infer that the LC states of TS1 and TS2, and the states of REF, record single molecules contacting the electrodes with their π structures. The trend of n (n_TS1(HC)_ < n_TS2(HC)_ < n_TS3_ and n_TS1(HC)_ < n_TS4_) correlates with that of the single‐molecule conductance (G_TS1(HC)_ > G_TS2(HC)_ > G_TS3_ and G_TS1(HC)_ > G_TS4_) (Figure 3b). In summary, the flicker noise analysis confirms that the electronic coupling and TSCT effect are determined by donor‐acceptor interactions, controlled by the electron deficiency of the acceptor group as well as by the relative through‐space distance and orientation between the donor and acceptor moieties. In particular, the flicker noise power of TS1 suggests ultra‐strong electronic coupling between the through‐space coupled D and A.

### 2.4 Junction Stretching Length Supports Ultra‐Strong Through‐Space Coupling

**FIGURE 4:**
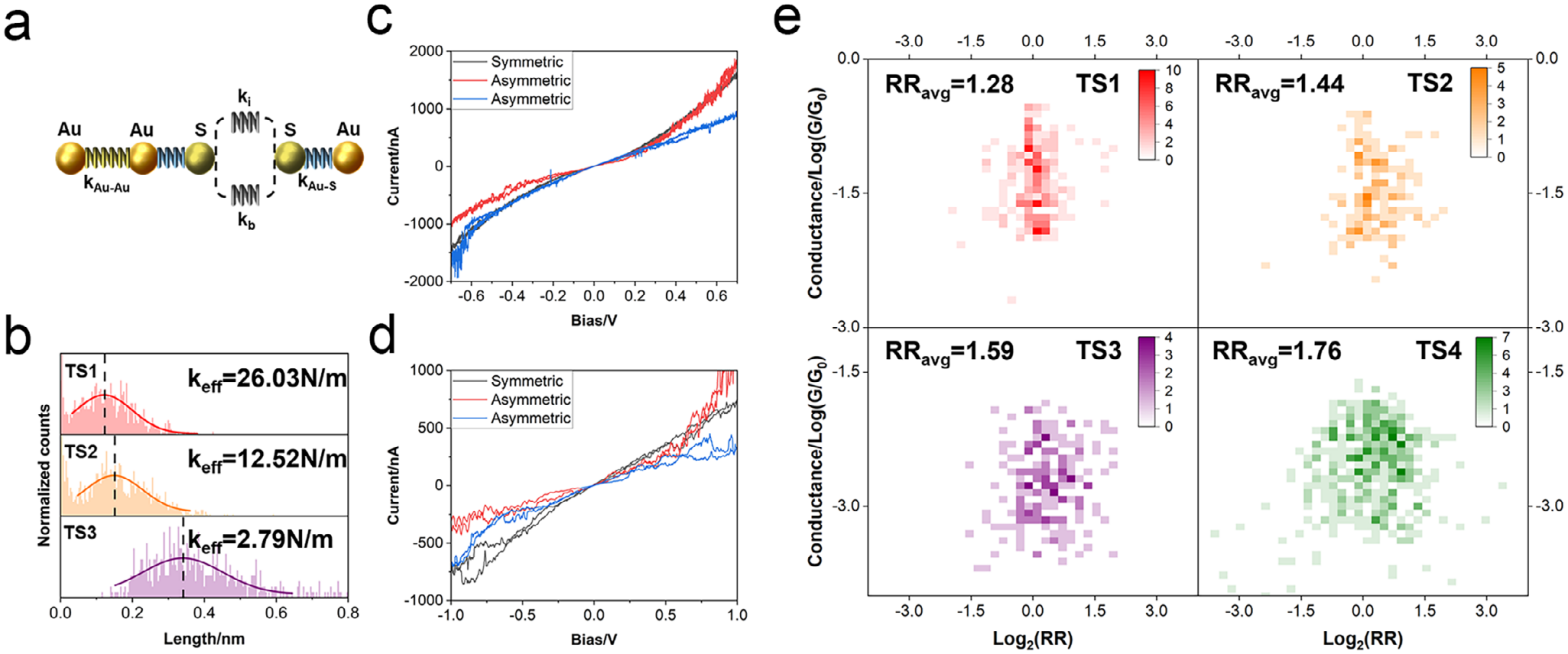
Mechanical and electronic characterization of the TSCT molecules. (a) Simplified spring model for the molecular junction. (b) Junction stretching length of TS1 (HC), TS2 (HC), and TS3. (c,d) Individual current‐bias voltage curves with different symmetries of TS1 (c) and TS4 (d). (e) 2D histograms of conductance vs. base‐2 logarithm of rectification ratio, Log_2_(RR), for TS1, TS2, TS3 and TS4. In this representation, a ratio of 1:1 corresponds to 0, and a ratio of 2:1 corresponds to 1, allowing the RR to be displayed more clearly.

The coupling strength between D and A is also reflected in the junction stretching length, i.e., the tip displacement from the junction formation to the breakdown. Longer stretching length indicates more flexible structures [23, 24]. We calculated the stretching length from junction formation to its breakdown based on the conductance‐distance traces (Supporting Information) and constructed 1D histograms of the stretching lengths measured. As shown in Figure 4, TS1(HC) and TS2(HC) exhibit similar stretching lengths, measuring 0.122 and 0.151 nm, respectively. In contrast, TS3 displays a larger stretching length of 0.346 nm (Figure 4b). Because the three molecules share the same bridge and anchoring groups, the main factor controlling junction flexibility is the strength of the through‐space interaction between D and A. The trend of junction stretching length (SL_TS1(HC)_ < SL_TS2(HC)_ < SL_TS3_) thus reflects a trend of D and A mechanical coupling strength that aligns with conductance results and flicker noise analysis. With the stretching length and the typical breakdown force of Au‐SMe [37], we estimated the spring constant of the D–A coupling based on a simplified model (Figure 4a; Table S2), and found a spring constant larger than 23.24 N/m. This value is comparable to that of the coordinate bond between the Fe atom and the Cp ring in a Fc molecule (23.3 N/m, stretching mode) [38]. We note that the value here represents a lower limit of the estimated spring constant of TS1, as factors such as rotational relaxation may also contribute to the recorded stretching distance. In future work, incorporating flexible PEO‐based linkers on both sides of the molecule and undertaking force‐spectroscopy measurements may help enable a more accurate determination of the molecular spring constant.

### 2.5 Current‐Voltage Spectroscopy Uncovers Strong TSCT Between Donor And Acceptor

To gain further insight into the interactions between the D and A subunits, we recorded current‐voltage (*I–V*) spectroscopy for TS1(HC), TS2(HC), TS3, and TS4. We scanned the tip‐substrate bias voltage between ±0.7 V for TS1(HC) and TS2(HC), and ±1 V for TS3 and TS4, while recording the current through the junction (Methods). A smaller bias range was used for TS1 and TS2, as the current in some *I–V* curves exceeded the upper detection limit (10 µA with a 1 µA/V current voltage amplifier) beyond ±0.7 V due to the high conductance of these two molecules (see Figure S20). Both symmetric and asymmetric individual *I–V* curves were observed across the four species (Figure 4c,d; Figure S20), with varying proportions of asymmetry. Statistical analysis of more than 170 *I–V* curves of TS1(HC) revealed an overall averaged rectification ratio (RR) of 1.28 at ±0.7 V (Figure 4e). In contrast, the averaged RR for TS4 was 1.76 (Figure 4e), with a broader distribution along the Log_2_(RR) axis. The insignificant current rectification of TS1 (HC) differs from that of single‐molecule diodes/rectifiers with covalently bound donor and acceptor subunits [22] but can be explained by the strong electronic coupling between D and A. The asymmetry in electronic structure is thus attenuated by the strong interaction between D and A, and the intramolecular partial charge transfer from D to A, resulting in a symmetric current‐voltage response. This is still further direct evidence of TSCT in the ground state of TS1. The increased average RR of TS4 confirms that an increase in the D–A distance decouples the subunits and attenuates the TSCT. This trend is further supported by the increasing rectification ratios observed for TS2(HC) and TS3, which are 1.44 and 1.59, respectively (Figure 4e). As the electronic coupling between the donor and acceptor weakens from TS1 to TS2 and further to TS3, the rectification ratio correspondingly increases.

The electron deficiency of the acceptor group also decreases along this series—potentially reducing the inherent asymmetry in the electronic structure—but this competing effect does not override the overall trend. We thus note that although TS3 contains two donor groups, these are not identical, and a slight asymmetry remains, likely contributing to the mild current rectification observed. The observed increased rectification ratio thus again supports that donor‐acceptor coupling strength is the dominant factor affecting current symmetry. The lack of rectification in TS1 is therefore primarily attributed to the significant partial TSCT in the ground state of the molecule.

### 2.6 Theoretical Calculations

**FIGURE 5:**
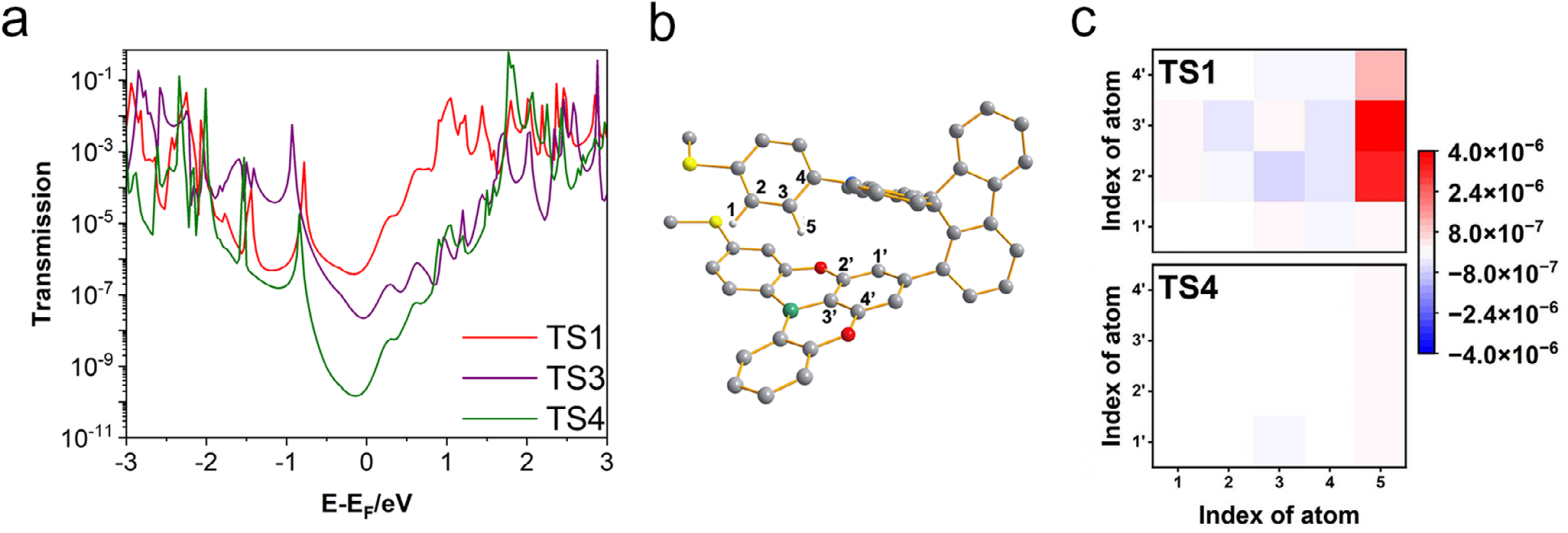
Theoretical simulations and transmission spectra of TS1, TS3, and TS4. (a) Calculated transmission spectra of a single TS1, TS3, and TS4 molecule sandwiched between two gold electrodes. (b) Ball‐and‐stick model of TS1 with atoms in the donor and acceptor subunits labeled as n(1‐5) and n‘(1‘‐4‘), respectively. The atom labeling of TS4 is the same as that of TS1; the only difference is that the two subunits are in a meta configuration. (c) Contributions of various atom pairs between the donor and acceptor subunits of TS1 (upper panel) and TS4 (lower panel) to the transmission coefficient of the molecular junctions at the Fermi level. For clarity, only the atom pairs with large contributions are shown. For TS4, the contributions from donor–acceptor atom pairs are negligible at the Fermi level and therefore appear very weak, resulting in a nearly blank and faintly colored panel.

A theoretical study of the charge transport properties of single TS1, TS3, and TS4 molecules sandwiched between two gold electrodes in vacuum using a combination of density functional theory (DFT) and the non‐equilibrium Green's function method was conducted [39]. The distances between electrodes were set according to the total tip displacements for each molecule (Figures S21 and S22). The simulation results presented in Figure 5a demonstrate that within the bias range of the experiments, the transmission of TS1 (red line) is significantly higher than for TS3 (purple line), followed by TS4 (green line), in good agreement with all the experimental data. We note in passing that the quantitative transmission values vary with the electrode separation used in the calculations, but this dependence does not affect the qualitative conclusions of the conductance comparison. The distinction between TS1 and TS4 hinges on the relative positioning of the donor and acceptor subunits. The donor and acceptor moieties in TS1 are in close proximity, whereas the two moieties in TS4 are so far apart that there is essentially no direct interaction. In contrast to TS4, where electrons are primarily transported via the donor‐bridge‐acceptor pathway, a new electron transport channel between the donor and acceptor subunits through a through‐space pathway is opened in TS1, which leads to a significant increase in the conductance. The new TS1 electron transport channel is strongly reflected in Figure 5c, which shows the contributions from various atom pairs between the donor and acceptor subunits to the transmission coefficient (Figure 5b). It is evident that for TS1, the charge transport from one of the hydrogen atoms (atom 5) on the donor to the three carbon atoms on the acceptor dominates (upper panel), while direct transport of electrons between the donor and acceptor subunits is negligible for TS4 (lower panel). Our simulations thus clearly demonstrate that through‐space charge transport is a key contributor to the observed high conductance of TS1.

## 3 Discussion

The nature of the strong through‐space coupling between the D and A moieties in the TS1 and TS2 members of the strategically chosen family of donor‐acceptor complexes observed in the present study differs from coupling through covalent chemical bonds but still exhibits extraordinarily high coupling strength, comparable to that of through‐bond coupling. First, flicker noise analysis discloses a G^1.2^ scaled PSD, resembling through‐bond coupling patterns. Second, the estimated spring constant of the D–A ‘through‐space bonding’ for TS1 exceeds 23.24 N/m, placing it within the regime of typical coordinate bonds, such as the bond between Fe and Cyclopentadienyl in Ferrocene (23.3 N/m, stretching mode [38]). Moreover, in the 2D nuclear magnetic resonance (NMR) spectrum of TS1 (Figure S1), the spatial interaction between D and A on TS1 shifts the i and h hydrogen atoms on the A unit to higher field due to the strong interaction [40] with D, analogous to the shifts of f and g hydrogen atoms in Tbyd (Figure S2), with a chemical bond directly connecting D and A. Previous single‐molecule electronic studies have reported enhanced interaction between π systems with dipole bearing groups [30] or rigid bridge groups [41], but none of these achieve the coupling strength and conductance observed in the present study.

A second notable observation is the exceptionally high single‐molecule conductance. The conductance of TS1, approximately 10^−0.71^G_0_, is nearly an order of magnitude higher than that of molecular conductive wires based on oligoynes [27] or metallapolynes [28] of similar lengths (or the extrapolation of the same length). The high conductance also reflects the strong coupling between the donor and acceptor. From an application aspect, the TSCT molecules are therefore novel and highly promising candidates for the construction of conductive molecular wires [42].

Nevertheless, the physical nature of the observed through‐space couplings differs fundamentally from coupling through covalent chemical bonds. The D–A through‐space coupling arises from the integrated non‐covalent, van der Waals‐like interaction between the delocalized orbitals rather than from overlapping and hybridization of atomic orbitals [43, 44]. In addition, a direct connection between D and A via conjugated bonds typically results in side‐by‐side overlapping of the orbitals, which often attenuates the light‐emitting functionality [45, 46]. In contrast, the through‐space coupling in TS1 features face‐to‐face orbital overlapping, providing multidimensional pathways for energy and charge‐transfer. As a result, rectification effects are absent, implying metal‐like rather than semiconductor‐like charge transport behavior. If this metal‐like through‐space charge transfer pathway were maintained in bulk solution, the luminescence properties observed should correlate with the strong D–A interaction. Future studies could focus on single‐molecule luminescence and on exploring also the potential of TSCT molecules as single‐molecule luminescent devices.

The high conductance observed for TS1 and TS2 thus originates from strong ground‐state through‐space charge transfer (TSCT), supported further by UV–vis absorption. Unlike established TSCT mechanisms that involve excited states that require high bias or photoexcitation, this ground‐state CT is non‐luminescent and without external excitation. However, based on concepts and formalism known for intervalence electronic transitions, the D–A charge transfer band would still enable calculating also the rate constant for the corresponding thermal D–A electron transfer rate constant, illuminating further the electronic conduction process [47, 48]. *I–V* spectroscopy further showed no conductance switching or bias‐induced transitions for TS1∼TS4 within the accessible bias ranges (±0.7 V for TS1 and TS2; ±1.0 V for TS3 and TS4) (Figure 4c,d; Figure S20c,d). Although these target molecules exhibit luminescence at higher excitation voltages (e.g., TS1 at ∼2.8 V), potentially linked to an excited‐state process involving the same donor‐acceptor interaction, this is beyond our current measurement range, as conductance saturates at higher voltages.

## 4 Conclusion

In summary, our study offers new direct and strong molecular‐level evidence that through‐space donor‐acceptor (TSDA) interactions can achieve electronic coupling strengths and charge transport efficiencies comparable to those of known through‐bond systems when optimized in terms of spatial alignment and electronic complementarity. Single‐molecule conductance discloses exceptionally high conductance, supported comprehensively by flicker noise analysis, mechanical recordings, and *I–V* characterization, all of which confirm the robustness of the through‐space D–A coupling. Comprehensive theoretical modeling and photophysical characterization further support through‐space transmission as the prevailing CT mechanism. These findings challenge broadly established views that covalent bonding is a prerequisite for efficient molecular conduction, and highlight TSDA architectures as a promising new platform for next‐generation organic electronic and optoelectronic devices.

## 5 Methods

### 5.1 Conductance Measurements

The conductance experiments were carried out using the scanning tunneling microscopy break‐junction (STM BJ) technique. Sample solutions were prepared by dissolving powders of the target molecules in pure mesitylene (TMB) to achieve a concentration of approximately 0.1 mM. Subsequently, a gold substrate was incubated with the solution for approximately 1 h, followed by rinsing with pure mesitylene. Assembly of the sample cell involved positioning the modified substrate and introducing pure mesitylene to establish a liquid environment. The STM tip was freshly cut from a gold wire. With bias set at 0.1 V, the tip was controlled to move repeatedly toward and away from the substrate. During the retraction of the tip, the conductance vs. tip displacement was recorded at a sampling rate of 10 kHz. All acquired curves exhibiting discernible plateaus were selected by a program with the same selection criteria, with over 10 % of the curves meeting the selection criteria. (Curves obtained from the blank (TMB) samples underwent the same selection process, with less than 0.2 % of the curves selected.) Finally, the selected curves with clear plateaus were compiled to construct both 2D and 1D histograms for further analysis.

### 5.2 Flicker Noise Analysis and *I–V* Characterization

The experimental procedures for flicker noise and *I–V* experiments are similar to those of the break‐junction procedure. Upon detection of a plateau by the program, the tip movement is halted for 100 ms, during which current was recorded at a sampling rate of 100 kHz. In the flicker noise experiment, the conductance vs. time was recorded with a bias set at 0.1 V. For the *I–V* experiments, a scanning bias voltage ranging from −0.7 to 0.7 V was applied on the electrodes for TS1, TS2, and −1.0 to 1.0 V on TS3 and TS4, during which the current against bias voltage was recorded for each curve. In the flicker noise analysis, a digital band‐pass filter of 100–1000 Hz was applied to pre‐process the experimental data to exclude the effects of low‐frequency vibrations and high‐frequency noise. The parts after the junction breakdown were excluded from the analysis. A discrete Fourier transformation on the data was then conducted, enabling the calculation of noise power through the integration of amplitude from 100 to 1000 Hz. For each individual trace, we normalized the noise power by the averaged conductance of the particular trace and constructed the 2D histograms of normalized flicker noise power against conductance. We calculated the Pearson correlation coefficients between normalized flicker noise power ((noise power)/G^n^) and the average conductance by increasing n from 1.0 to 2.0 with a 0.01 gradient; then the scaling exponents were determined to be the values of n that correspond to the smallest absolute values of the correlation coefficients. We used an applied 2D Gaussian distribution for fitting to each 2D histogram, the black ellipses representing contour lines of the fitted 2D Gaussian distribution equation. *I–V* current‐voltage data was recorded. Each complete *I–V* curve was used to construct a 2D histogram of current vs. voltage. The rectification ratio for each curve was calculated by the ratio of current at V_bias_ = ±0.7 V. By combining the rectification ratio and conductance of each curve, a 2D histogram was generated to analyze the *I–V* symmetry for each molecule. The average rectification ratio was computed by taking the rectification ratio, I_positive_/|I_negative_|, of each individual curve at ±0.7 V, using the original RR for forward rectification and the reciprocal for reverse rectification, and dividing the sum by the total number of curves.

### 5.3 Theoretical Calculations

All DFT calculations were carried out using QuantumATK [49]. The FHI pseudopotentials were used to describe interactions between ionic cores and valence electrons [50]. The Perdew–Burke–Ernzerhof (PBE) functional was employed to describe the correlation‐exchange interaction [51], and the dispersion interaction was included by using the DFT‐D3 method of Grimme with a zero‐damping function [52]. The double‐ζ plus single polarization basis sets (DZP) were used to expand the wave functions. The lattice constant of Au was optimized to 4.118 Å, in good agreement with previous calculations [53]. The two metal electrodes were modeled by two 3‐layer Au (111) surfaces, each with a pyramidal Au_20_ cluster on top. A molecule in the middle binds to the two Au_20_ clusters (Figure S22: the region between the two red vertical lines). An 8 × 8 supercell was used for the Au (111) surface in each of the electrode models. The Brillouin zone was sampled by using the Γ point only. The distance between the two electrodes was defined as the projection of the distance between the two sulfur atoms at each end of the central molecule along the direction of electron transport, with the specific values derived from experimental data. The bottom two atomic layers in each Au electrode were kept frozen during atomic relaxation, while all other atoms, including those in the central molecule, were relaxed until the maximum force was less than 0.05 eV/Å.

Based on the configuration obtained, a molecular junction model was constructed to calculate the electron transport properties by adding seven additional Au(111) atomic layers for each Au electrode (Figure S22), three of which serve as the bulk electrode. The simulations were carried out by combining DFT with the non‐equilibrium Green's function (NEGF) method [39]. The same functional, pseudopotential, and basis sets were used as for the structure optimization, except that for Au atoms, the single‐ζ plus single polarization basis sets (SZP) were used to reduce the computational cost. The 1 × 1 × 133 Monkhorst–Pack grids were used for the transport calculations and the 3 × 3 Monkhorst–Pack grids for the transmission spectrum and pathway analysis [54]. The electron temperature was set to 1000 K, and the real‐space density mesh cutoff to 75 Hartree. The imaginary part used in the Green's function calculations was set to 10^−6^ eV. All theoretical calculations refer to the molecular junction in vacuum, warranted by the apolar nature of the mesitylene solvent.

### 5.4 Absorption, Steady‐State PL Spectra and PLQE Measurements

UV–vis absorption spectra were recorded using a Shimadzu UV‐3600 spectrophotometer. Fluorescence spectra were recorded using a Hitachi F‐4600 spectrophotometer. Absolute PL quantum efficiencies (PLQEs) were calculated by a FLS1000 photoluminescence spectrometer. The transient photoluminescence (PL) decay curves were obtained by FluoTime 300 (PicoQuant GmbH) with a Picosecond Pulsed UV‐LASTER (LASTER375) as the excitation source.

## Funding

National Key Research and Development Program of China (2021YFA1200101) (Y. Li), National Key Research and Development Program of China (2021YFA1200104) (J. Li), National Natural Science Foundation of China (22174134) (Y. Li), National Natural Science Foundation of China (22273091) (Q. Fu), National Natural Science Foundation of China (52103242) (L. Cui), National Natural Science Foundation of China (22474133) (Y. Li), C.A.S Project for Young Scientists in Basic Research (YSBR‐054) (Y. Li).

## Conflicts of Interest

The authors declare no conflict of interest.

## Supporting information




**Supporting File**: advs73600‐sup‐0001‐SuppMat.docx.

## Data Availability

The data that support the findings of this study are available in the supplementary material of this article.
