# Supplementary material for: Probing Ultrastrong Through‐Space Electronic Coupling in Donor‐Acceptor Systems at the Single‐Molecule Level
Source: Adv Sci (Weinh). 2026 Jan 4;13(14):e21879. doi: 10.1002/advs.202521879 (PMC12970254; doi:10.1002/advs.202521879)
Supplement: Supplementary file 1 — Supporting File: advs73600‐sup‐0001‐SuppMat.docx. [file ADVS-13-e21879-s001.docx]

Supporting Information

Probing ultrastrong through-space electronic coupling in donor-acceptor systems at the single-molecule level

Xin Wang, Dan Yang, Jiazheng Diao, Jens Ulstrup, Chengyang Zhang, Florian Auras, Qiang Fu*, Jinlong Yang, Linsong Cui*, Yueqi Li* & Jinghong Li

**Supporting Text**

Plateau selection criteria

All data were recorded using the approach described in the main text. The tip was retracted at a rate of 40 nm/s. For all curves, plateaus were grouped into the high-conductance state if the conductance change (Log(ΔG/G_0_)) was less than 0.04 order of magnitude within 2 ms time duration and 10^-0.5^G_0_ to 10^-1.5^G_0_ conductance range. Plateaus were grouped into the low-conductance state if the conductance change (Log(ΔG/G_0_)) was less than 0.12 order of magnitude within 3 ms time duration and 10^-2.0^G_0_ to 10^-4.0^G_0_ conductance range. The junction formation yields were significantly higher for the target molecules than for the pure solvent (mesitylene), and amount to 11.73%, 6.89%, 4.30%, 5.89%, 12.61%, 14.71%, 27.17%, 48.43% and 72.38% for TS1, TS2, TS3, TS4, TS5, REF, 1,2-BDT, 1,3-BDT and 1,4-BDT while only 0.64% for mesitylene. The total number of conductance-distance traces collected for each molecule is as follows: TS1 (6600), TS2 (17921), TS3 (18430), TS4 (4453), TS5 (5527), REF (6450), 1,2-BDT (1001), 1,3-BDT (830), 1,4-BDT (3327) and mesitylene (5018).

Junction stretching length, total tip displacement and slope

We obtained the standard deviation (σ) of single-molecule conductance from Gaussian fitting of the peaks in 1D conductance histograms. Then we calculated the junction stretching length from the formation (G+3σ) to the breakdown (G-3σ) of molecular junction in each individual conductance decay curve. We conducted a statistical analysis to obtain the junction stretching length for each plateau. Similarly, we analyzed the conductance plateau slopes by fitting the conductance decay G+σ to G-σ in each individual curve. Total tip displacement was calculated from the breakdown of gold-gold point contact (G_0_) to the breakdown of junction (G-3σ) plus snap-back distance (0.5 nm). A narrower range was chosen for the calculation of plateau slope than for the stretching length to avoid error from the pre-plateau and post-plateau sharp decay on the curves.

Spring Constants

In the estimation of spring constants, we built a simplified model to intuitively treat the single-molecule junction as springs in series. In the middle of the junction, the D-B-A moiety is analogous to two springs in parallel (Figure 3a). One spring is attributed to the through-space donor-acceptor interaction (spring constant defined as k_i_), the other spring roughly describes the constraint from the backbone (spring constant defined as k_b_). k_i_ is different for different acceptor moieties. We took k_b_ to be approximately the same for TS1, TS2 and TS3. We obtained the force of the junctions at breakdown, F, from the reported breakdown force of the Au-SMe bond (~0.7 nN). With the spring constants of the Au-Au bond (8 N/m), S-Au bond (184 N/m) and junction stretching length (SL), we calculated the proportion of stretching distance allocated on the D-B-A moiety, Δx, for each target molecule as,

$$\frac{\text{F}}{\text{k}_{\text{Au-Au}}}\text{+}\frac{\text{2F}}{\text{k}_{\text{Au-S}}}\text{+}\text{Δ}\text{x = SL}$$

We calculated the “effective” spring constant, $\text{k}_{\text{eff}}$, of the D-B-A moiety from

$$\text{k}_{\text{eff}}\text{=}\frac{\text{F}}{\text{∆x}}$$

where $\text{k}_{\text{eff}}\text{ =}\text{ k}_{\text{b}}\text{ + }\text{k}_{\text{i}}$. Since ${\text{k}\text{ }}_{\text{i}}\text{≥}\text{ }\text{0}$, so $k_{b}\leq k_{\mathrm{eff}}$. With $\text{k}_{\text{eff}}$ of TS1, TS2 and TS3, we obtain $\text{k}_{\text{b}}\text{ ≤ 2.79 N}\text{/}\text{m}$. The lower limit of k_i_ for TS1 is $\mathrm{therefore} \text{23.24 N/m}$.

Synthesis and Characterization

The experimental details on the synthesis of TS1, TS2, TS3, TS4, TS5 and REF are summarized in the Scheme S1. ^1^H NMR and ^13^C NMR spectra in CDCl_3_ or DMSO-d_6_ solutions were recorded using a Bruker Biospin Avance-III 400 MHz NMR or a JEOL JNM-ECP600 spectrometer at 298K with chemical shifts (δ, ppm) relative to tetramethyl silane (Me4Si) for the ^1^H NMR and ^13^C NMR spectra.^1^H-^1^H COSY, HSQC and HMBC NMR (600 MHz) spectra in CDCl_3_ were recorded using a JEOL JNM-ECP600 spectrometer at 298K.


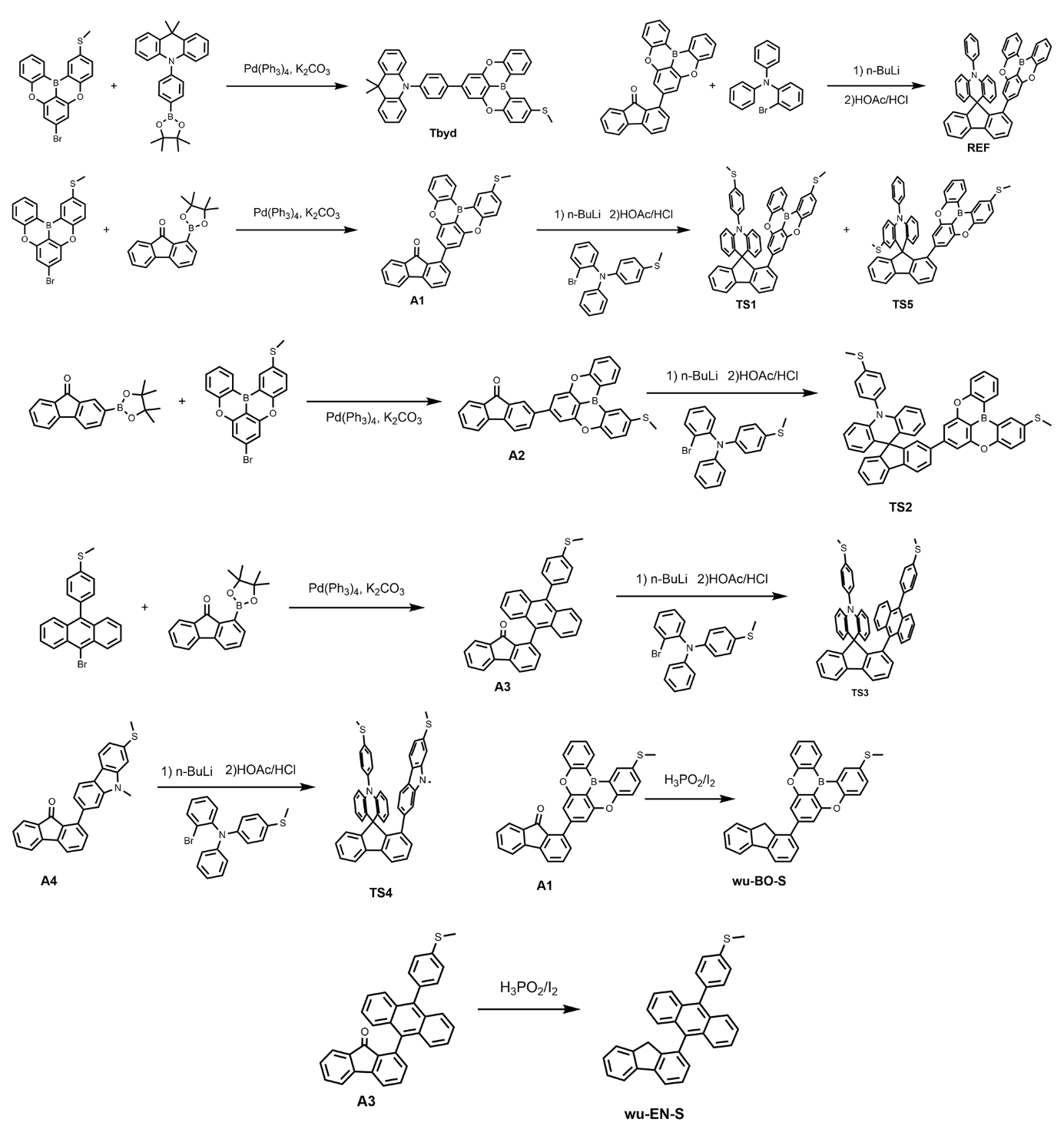


**Scheme S1.** Synthesis routes of TSn (n=1~5), REF, Tbyd, wu-BO-S and wu-En-S.

**Tbyd:**

1 (0.1g, 0.25 mmol), 2 (0.11g, 0.27 mmol), Pd (PPh_3_)_4_ (8 mg, 0.007 mmol) and potassium carbonate (0.07 g, 0.5 mmol) were dissolved in a two-neck flask, in 10 mL THF and 2 mL water under nitrogen. The mixture was stirred and heated at 80 °C for 24 h. After cooling to room temperature, the mixture was concentrated under reduced pressure to remove THF and then extracted by DCM (3 x 8 mL). The crude product was purified by column chromatography on silica gel using petroleum-dichloromethane (4:1) to obtain Tbyd as a yellow powder (0.095 g, 62.6%).

**REF:**

2-bromo triphenylamine (0.09 g, 0.27 mmol) was dissolved in 25 mL dehydrated THF in a two-neck flask, under nitrogen. The mixture was cooled to -78 °C for 10 minutes. 2.5 M n-BuLi (0.12 mL, 0.29 mmol) was then added drop-wise over 10 minutes and the mixture stirred at -78 °C for 1 h. 3(0.1 g, 0.22 mmol) was then added and the mixture stirred at room temperature overnight. 10 mL distilled water was added to quench the reaction. The mixture was concentrated under reduced pressure to remove THF and then extracted by DCM (3 x 8 mL). The DCM was removed under reduced pressure and the solid recrystallized from 25 mL ethyl acetate. After filtration, the residue was dissolved in 3 mL HOAc and 0.3 mL HCl added. The mixture was stirred at 110 °C for 3 h. After cooling to room temperature, the mixture was poured into 20 mL ice water and filtered to obtain the crude product. The crude product was purified by column chromatography on silica gel using petroleum-dichloromethane (4:1) to obtain REF as a white powder (0.1 g, 66.35%).

**A1:**

1 (0.4 g, 1.01 mmol), 3 (0.371 g, 1.21 mmol), Pd (PPh_3_)_4_ (10 mg, 0.01 mmol) and potassium carbonate (0.28 g, 2.02 mmol) were dissolved in 15 mL THF and 5 mL water in a two-neck flask, under nitrogen. The mixture was stirred and heated at 80°C for 7 h. After cooling to room temperature, the mixture was concentrated under reduced pressure to remove THF and then extracted by DCM (3×10 mL). The crude product was recrystallized from n-hexane and dichloromethane to obtain a yellow solid (0.377 g, 75.32%).

**TS1 and TS5:**

2-bromo-N-(4-(methylthio)phenyl)-N-phenylaniline (0.27 g, 0.73 mmol) was dissolved in 25 mL dehydrated THF in a two-neck flask, under nitrogen. The mixture was cooled to -78 °C for 10 minutes. 2.4 M n-BuLi (0.33 mL, 0.79 mmol) was then added drop-wise over 10 minutes and the mixture stirred at -78 °C for 1 h. A1(0.3 g, 0.61 mmol) was then added and the mixture stirred at room temperature overnight. 10 mL distilled water was added to quench the reaction. The mixture was concentrated under reduced pressure to remove THF and then extracted by DCM (3 x 5 mL). DCM was removed under reduced pressure and the solid recrystallized from 10 mL n-hexane. After filtration, the residue was dissolved in 5 mL HOAc and 1 mL HCl added. The mixture was stirred at 95 °C for 3 h. After cooling to room temperature, the mixture was poured into 20 mL ice water and filtered to obtain the crude product. The crude product was purified by column chromatography on silica gel using petroleum-dichloromethane (5:1) to obtain TS1(0.12 g, 25.76%) and TS5(0.2 g, 42.93%) as a yellow powder.

**TS4:**

2-bromo-N-(4-(methylthio)phenyl)-N-phenylaniline (0.27 g, 0.73 mmol) was dissolved in 25 mL dehydrated THF in a two-neck flask, under nitrogen. The mixture was cooled to -78 °C for 10 minutes. 2.4 M n-BuLi (0.33 mL, 0.79 mmol) was added drop-wise over 10 minutes and the mixture stirred at -78 °C for 1 h. A1(0.3 g, 0.61 mmol) was added and the mixture stirred at room temperature overnight. 10 mL distilled water was then added to quench the reaction. The mixture was concentrated under reduced pressure to remove THF and then extracted by DCM (3 x 5 mL). DCM was removed under reduced pressure and the solid recrystallized from 10 mL n-hexane. After filtration, the residue was dissolved in 5 mL HOAc and 1 mL HCl added. The mixture was stirred at 95 °C for 3 h. After cooling to room temperature, the mixture was poured into 20 mL ice water and filtered to obtain the crude product. The crude product was purified by column chromatography on silica gel using petroleum-dichloromethane (5:1) to obtain TS4(0.19 g, 40.8%) as a white powder.

**A3:**

3 (0.48 g, 1.58 mmol), 4 (0.5 g, 1.32 mmol), Pd (PPh_3_)_4_ (13.6 mg, 0.013 mmol) and potassium carbonate (0.36 g, 2.64 mmol) were dissolved in 20 mL THF and 7 mL water in a two-neck flask, under nitrogen. The mixture was stirred and heated at 80 °C for 7 h. After cooling to room temperature, the mixture was concentrated under reduced pressure to remove THF and then extracted by DCM (3×10 mL). The crude product was recrystallized from n-hexane and dichloromethane to obtain A3 as a yellow solid (0.48 g, 76.08%).

**TS2:**

2-bromo-N-(4-(methylthio)phenyl)-N-phenylaniline (0.37 g, 1 mmol) was dissolved in 25 mL dehydrated THF in a two-neck flask, under nitrogen. The mixture was cooled to -78 °C for 10 minutes. 2.4 M n-BuLi (0.45 mL, 1.09 mmol) was then added drop-wise over 10 minutes and the mixture stirred at -78 °C for 1 h. A3(0.4 g, 0.84 mmol) was then added and the mixture stirred at room temperature overnight. 10 mL distilled water was added to quench the reaction. The mixture was concentrated under reduced pressure to remove THF and then extracted by DCM (3 x 10 mL). DCM was removed under reduced pressure and the solid recrystallized from 10 mL n-hexane. After filtration, the residue was dissolved in 6 mL HOAc and 1.2 mL HCl added. The mixture was stirred at 95 °C for 3 h. After cooling to room temperature, the mixture was poured into 20 mL ice water and filtered to obtain the crude product. The crude product was purified by column chromatography on silica gel using petroleum-dichloromethane (5:1) to get TS2(0.23 g, 36.6%) as a yellow powder.

**TS3:**

2-bromo-N-(4-(methylthio)phenyl)-N-phenylaniline (0.11 g, 0.3 mmol) was dissolved in 25 mL dehydrated THF in a two-neck flask, under nitrogen. The mixture was cooled to -78 °C for 10 minutes. 2.4 M n-BuLi (0.14 mL, 0.32 mmol) was then added drop-wise over 10 minutes and the mixture stirred at -78 °C for 1 h. A4 (0.1 g, 0.25 mmol) was then added and the mixture stirred at room temperature overnight. 10 mL distilled water was added to quench the reaction. The mixture was concentrated under reduced pressure to remove THF and then extracted by DCM (3 x 5 mL). DCM was removed under reduced pressure and the solid recrystallized from 10 mL n-hexane. After filtration, the residue was dissolved in 2 mL HOAc and 0.4 mL HCl added. The mixture was stirred at 95 °C for 3 h. After cooling to room temperature, the mixture was poured into 20 mL ice water and filtered to obtain the crude product. The crude product was purified by column chromatography on silica gel using petroleum-dichloromethane (5:1) to obtain TS3(0.07 g, 41.8%) as a brown powder.

**wu-BO-S:**

Iodine (0.05 g, 0.2 mmol) and acetic acid (5 mL) were added to a two-neck flask under nitrogen and stirred. 0.106 ml (0.81 mmol) of 50% aq hypophosphite was then added and the mixture heated to 100 °C. A1 (0.2 g, 0.4 mmol) in acetic acid solution (2 mL) was added to the reaction mixture and refluxed for 12 hours. After cooling to room temperature, the mixture was poured into 20 mL ice water and filtered to obtain the crude product. The crude product was purified by column chromatography on silica gel using petroleum-dichloromethane (5:1) to obtain wu-BO-S (0.14 g, 72.04%) as a white powder.

**wu-EN-S:**

Iodine (0.06 g, 0.21 mmol) and acetic acid (5 mL) were added to a two-neck flask under nitrogen and stirred. 0.106 ml (0.81 mmol) of 50% aq hypophosphite was then added and the mixture heated to 100 °C. A3 (0.2 g, 0.42 mmol), and acetic acid solution (2 mL) added to the reaction mixture and refluxed for 12 hours. After cooling to room temperature, the mixture was poured into 20 mL ice water and filtered to obtain the crude product. The crude product was purified by column chromatography on silica gel using petroleum-dichloromethane (5:1) to get wu-EN-S (0.17 g, 87.56%) as a white powder.

**Supporting Figure**


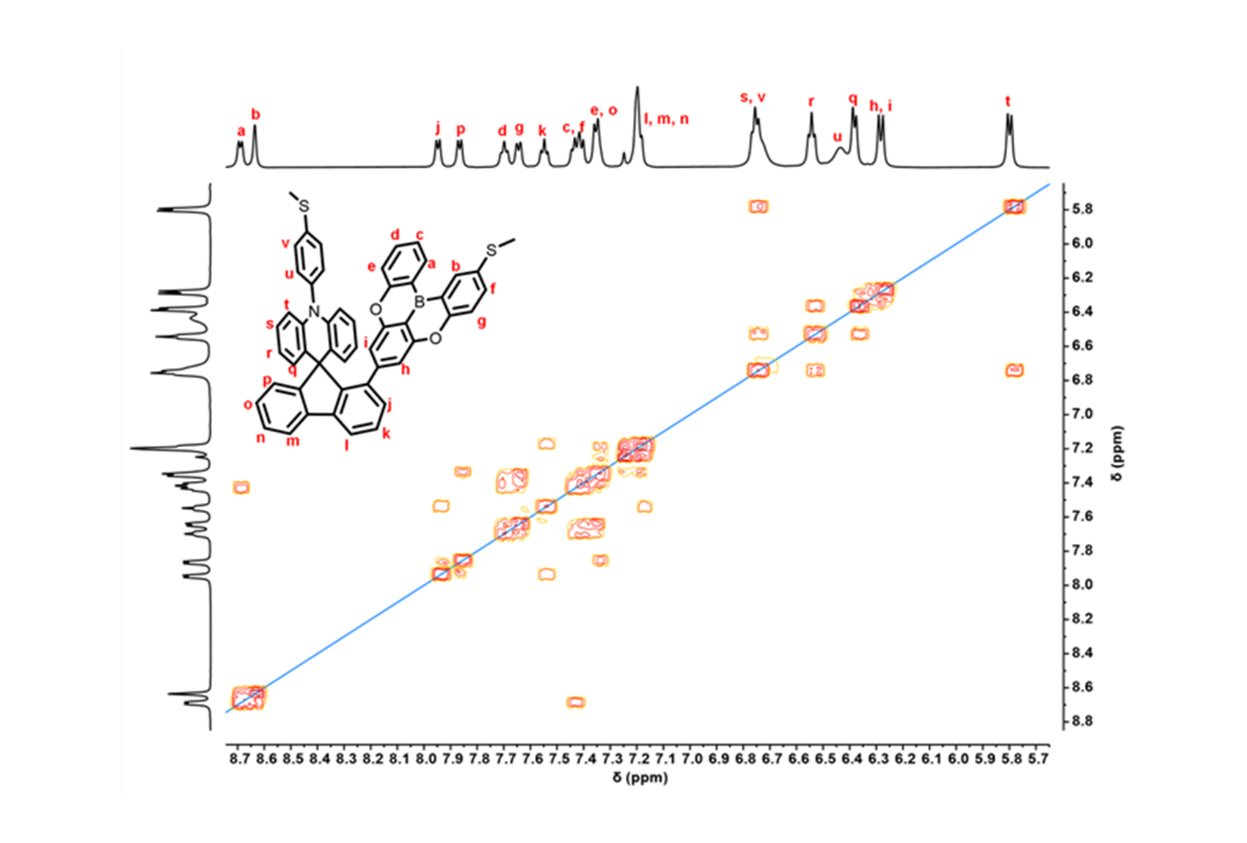


**Figure S1.** Partial ^1^H-^1^H COSY NMR (600 MHz) spectrum of TS1 at room temperature.


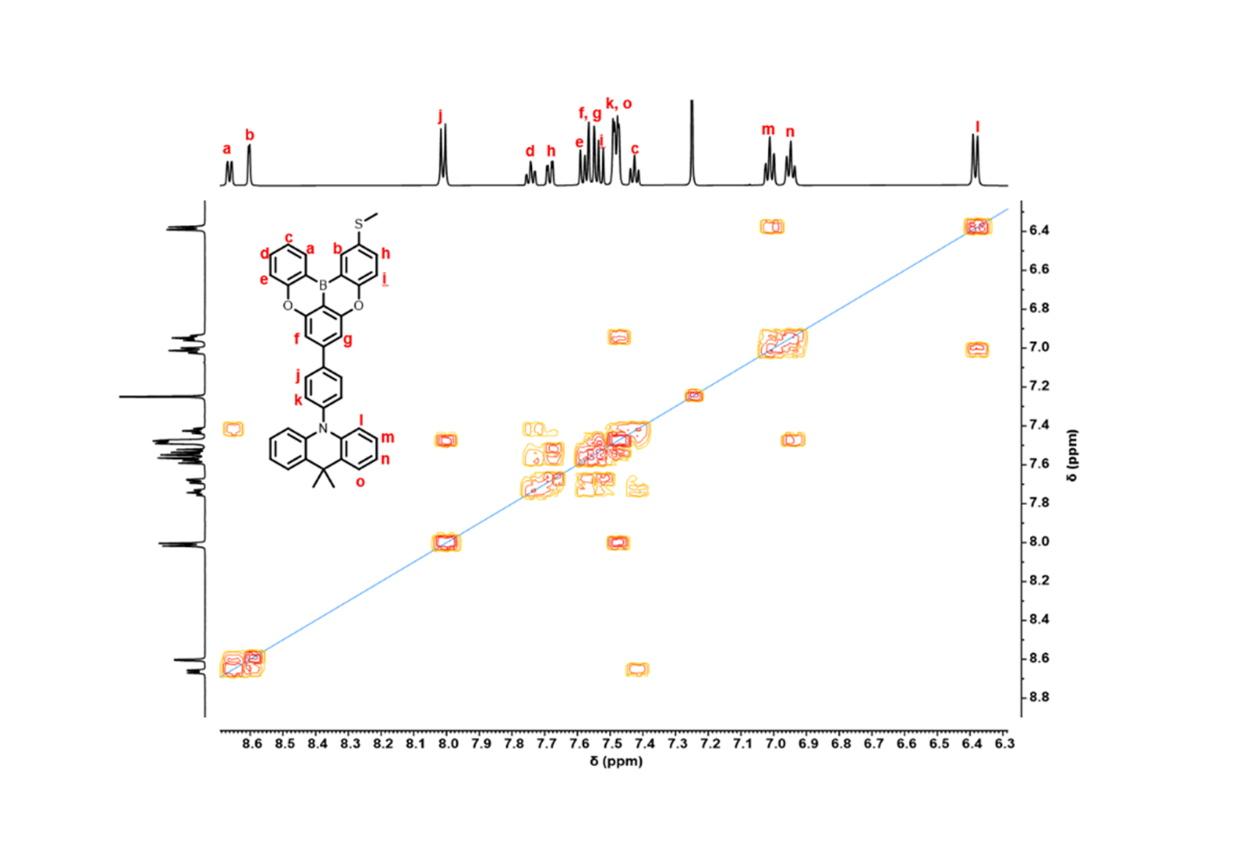


**Figure S2.** Partial ^1^H-^1^H COSY NMR (600 MHz) spectrum of Tbyd at room temperature.

**
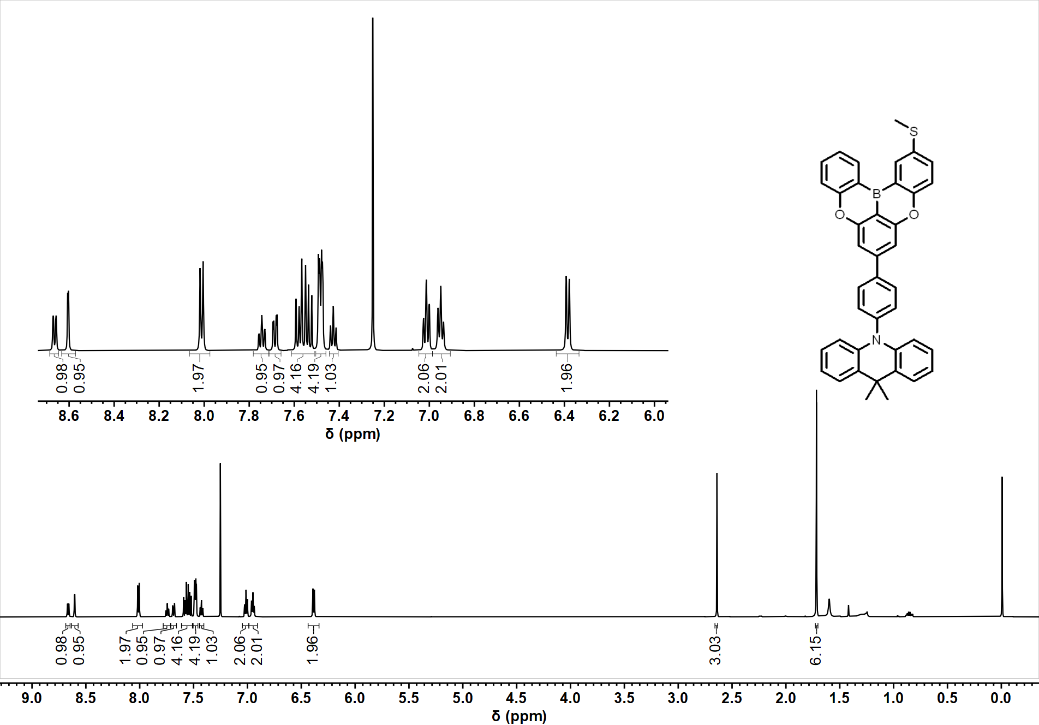
**

**
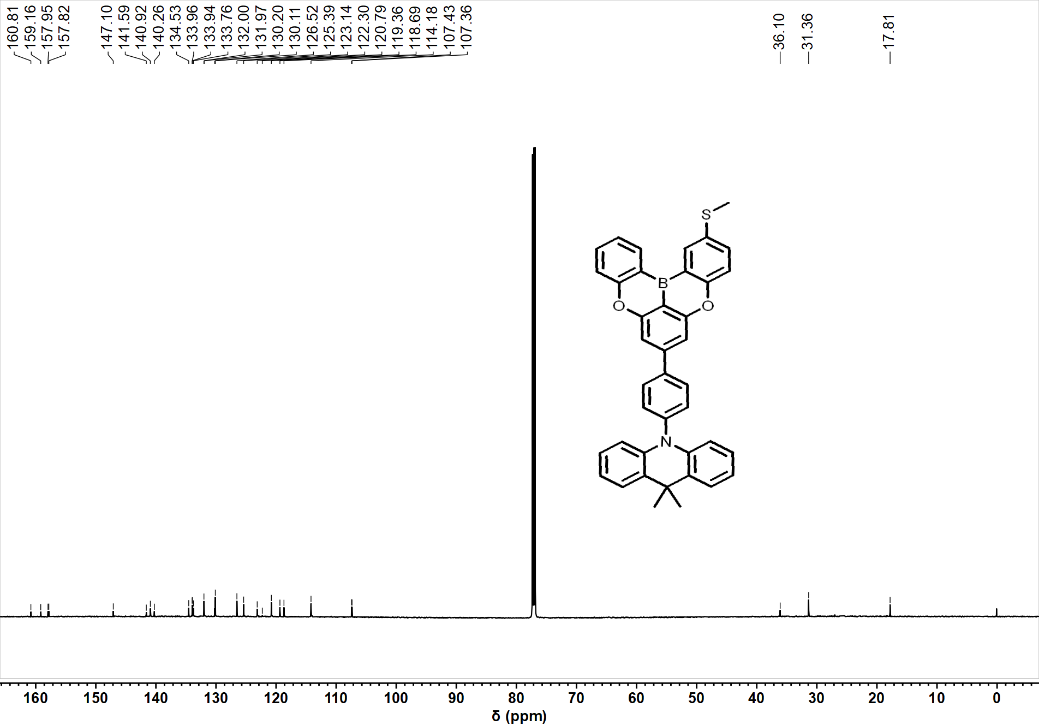
**

**Figure S3.** ^1^H NMR (top) and ^13^C NMR (bottom) spectra of Tbyd in CDCl_3_ at room temperature. ^1^H NMR (600 MHz, Chloroform-d) δ 8.66 (dd, J = 7.7, 1.7 Hz, 1H), 8.60 (d, J = 2.4 Hz, 1H), 8.03 – 7.98 (m, 2H), 7.74 (ddd, J = 8.5, 7.0, 1.6 Hz, 1H), 7.69 (dd, J = 8.7, 2.3 Hz, 1H), 7.60 – 7.51 (m, 4H), 7.51 – 7.46 (m, 4H), 7.44 – 7.41 (m, 1H), 7.01 (ddd, J = 8.5, 7.1, 1.5 Hz, 2H), 6.95 (t, J = 7.4 Hz, 2H), 6.38 (d, J = 8.1 Hz, 2H), 2.64 (s, 3H), 1.72 (s, 6H). ^13^C NMR (151 MHz, Chloroform-d) δ 160.81, 159.16, 157.95, 157.82, 147.10, 141.59, 140.92, 140.26, 134.53, 133.96, 133.94, 133.76, 132.00, 131.97, 130.20, 130.11, 126.52, 125.39, 123.14, 122.30, 120.79, 119.36, 118.69, 114.18, 107.43, 107.36, 36.10, 31.36, 17.81.

**
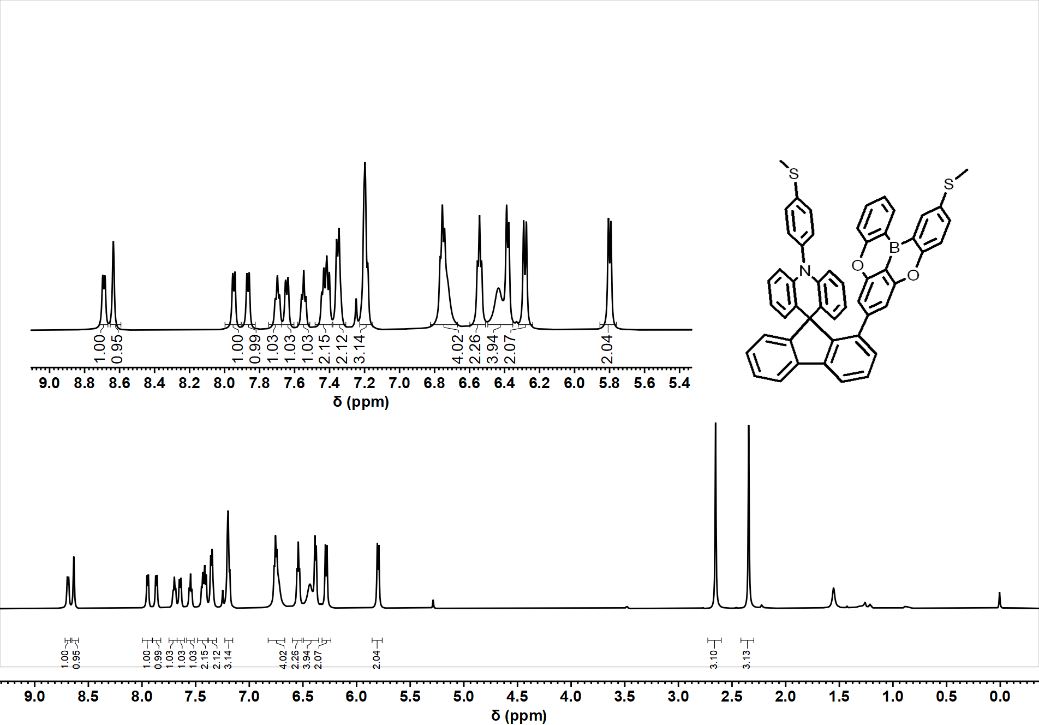
**

**
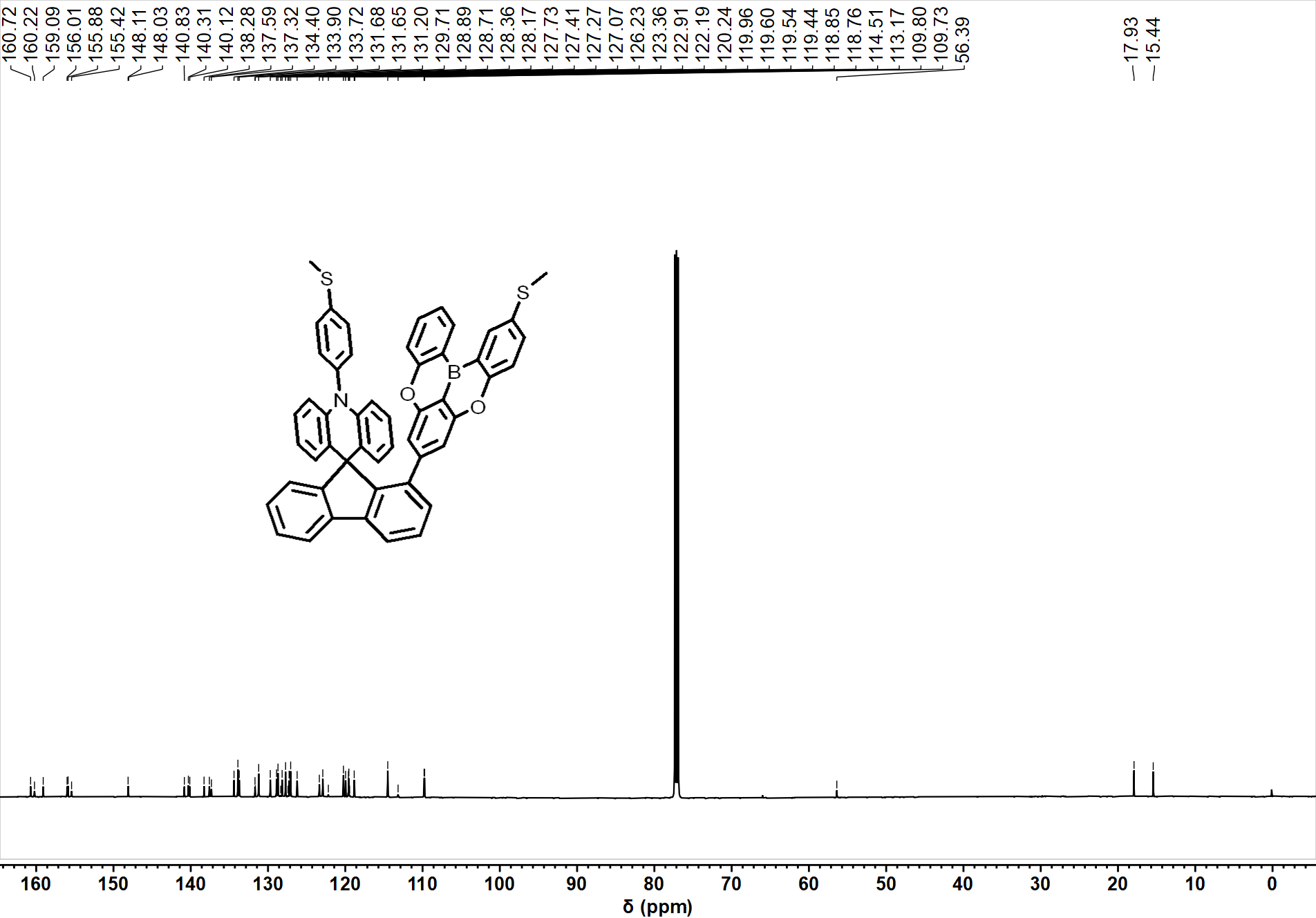
**

**Figure S4.** ^1^H NMR (top) and ^13^C NMR (bottom) spectra of TS1 in CDCl_3_ at room temperature. ^1^H NMR (600 MHz, Chloroform-d) δ 8.69 (d, J = 7.7 Hz, 1H), 8.63 (s, 1H), 7.94 (d, J = 7.6 Hz, 1H) , 7.86 (d, J = 7.7 Hz, 1H), 7.70 (t, J = 7.8 Hz, 1H), 7.64 (d, J = 8.6 Hz, 1H), 7.54 (t, J = 7.7 Hz, 1H), 7.50 – 7.38 (m, 2H), 7.35 (d, J = 9.0 Hz, 2H), 7.19 (q, J = 6.8, 6.0 Hz, 3H), 6.74 (q, J = 14.0, 10.9 Hz, 4H), 6.53 (t, J = 7.4 Hz, 2H), 6.39 (t, J = 17.7 Hz, 4H), 6.27 (d, J = 8.9 Hz, 2H), 5.79 (d, J = 8.4 Hz, 2H), 2.65 (s, 3H), 2.34 (s, 3H). ^13^C NMR (151 MHz, Chloroform-d) δ 160.72, 160.22, 159.09, 156.01, 155.88, 155.42, 148.11, 148.03, 140.83, 140.31, 140.12, 138.28, 137.59, 137.32, 134.40, 133.90, 133.72, 131.68, 131.65, 131.20, 129.71, 128.89, 128.71, 128.36, 128.17, 127.73, 127.41, 127.27, 127.07, 126.23, 123.36, 122.91, 122.19, 120.24, 119.96, 119.60, 119.54, 119.44, 118.85, 118.76, 114.51, 113.17, 109.80, 109.73, 56.39, 17.93, 15.44.


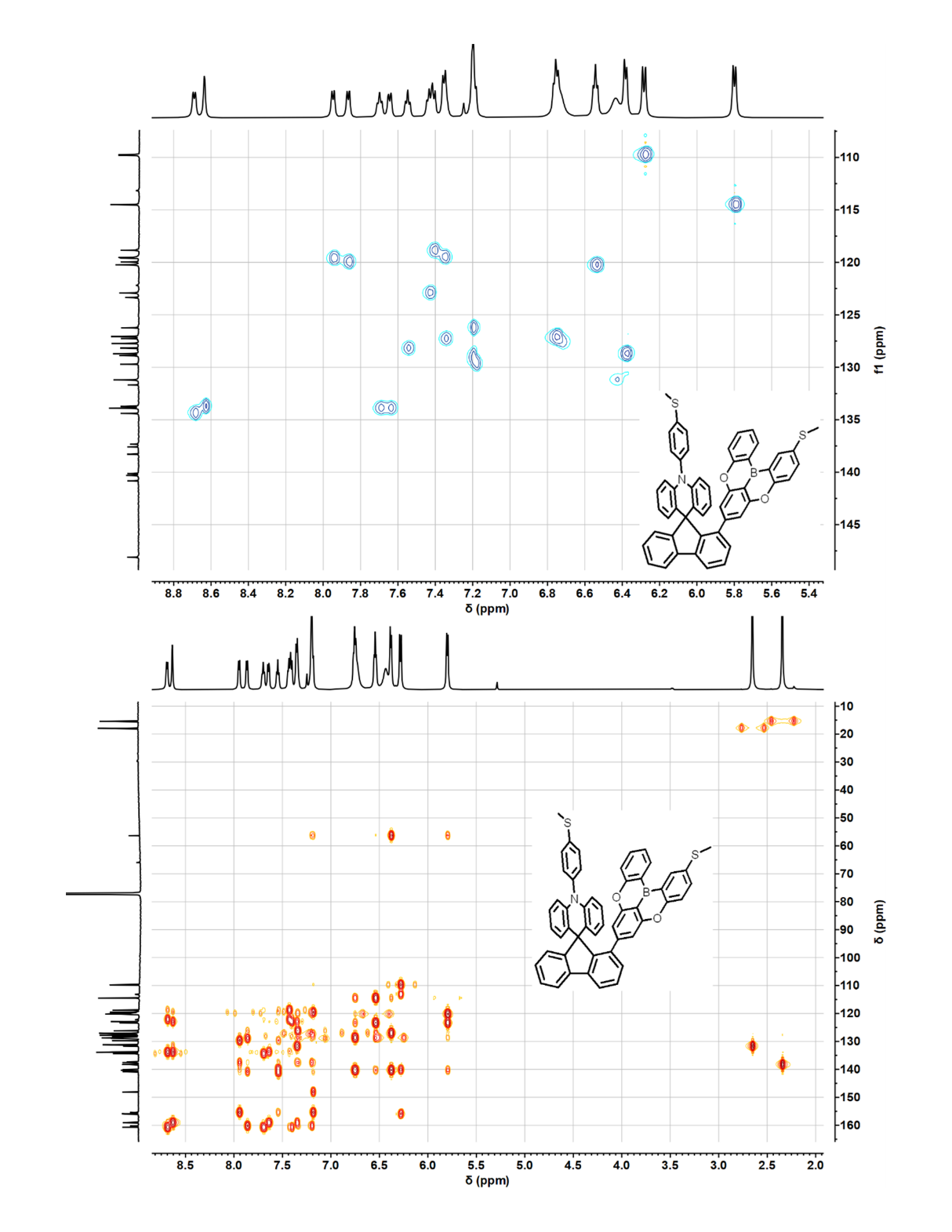


**Figure S5.** ^1^H NMR, ^13^C NMR, heteronuclear singular quantum correlation (HSQC), heteronuclear multiple bond correlation (HMBC) spectra of TS1 in CDCl_3_ at room temperature.

**
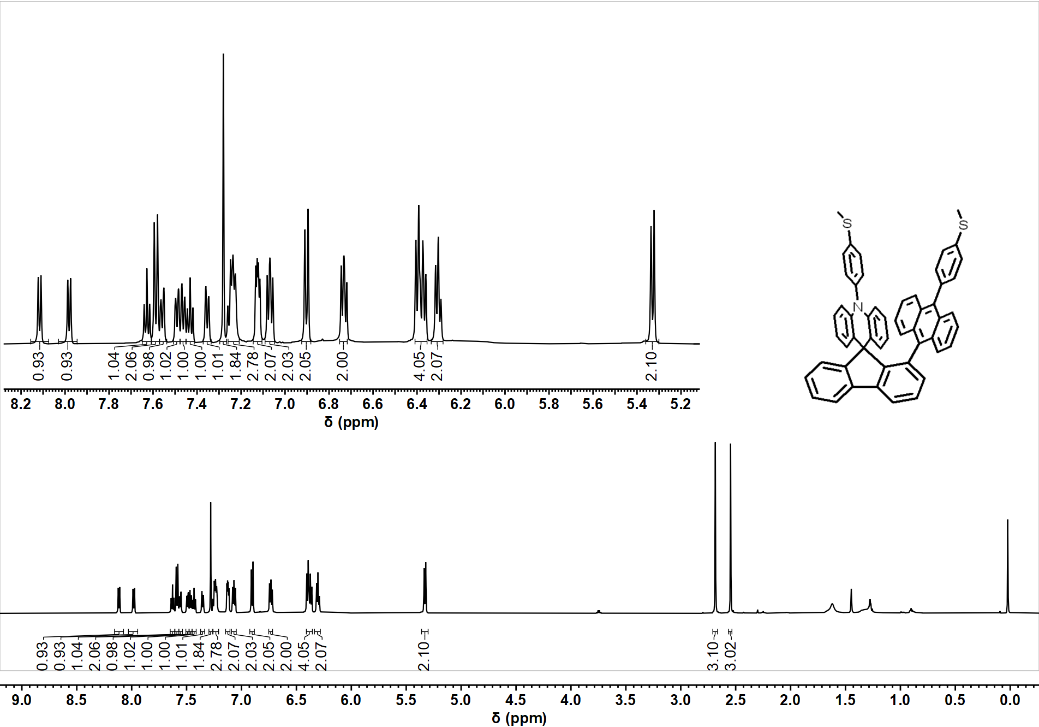
**

**
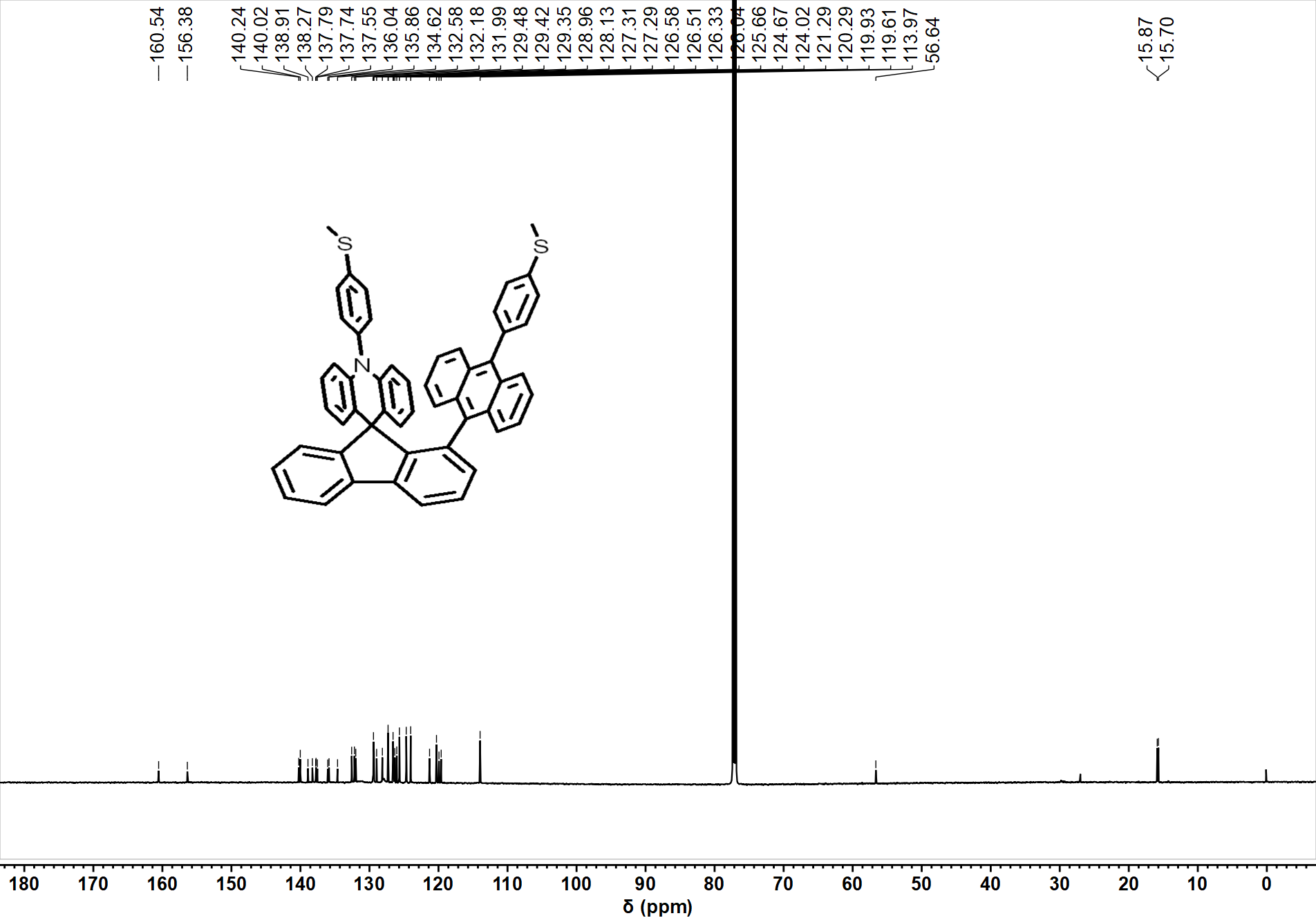
**

**Figure S6.** ^1^H NMR (top) and ^13^C NMR (bottom) spectra of TS2 in CDCl_3_ at room temperature. ^1^H NMR (600 MHz, Chloroform-d) δ 8.08 (d, J = 7.6 Hz, 1H), 7.95 (d, J = 7.7 Hz, 1H), 7.60 (t, J = 7.5 Hz, 1H), 7.56 (d, J = 8.9 Hz, 2H), 7.53 (dd, J = 8.0, 2.1 Hz, 1H), 7.45 (ddd, J = 16.8, 7.9, 2.0 Hz, 2H), 7.40 (t, J = 7.5 Hz, 1H), 7.32 (dd, J = 7.8, 1.9 Hz, 1H), 7.28 (s, 2H)7.21 (dd, J = 7.9, 5.2 Hz, 3H), 7.09 (dd, J = 7.5, 4.0 Hz, 2H), 7.07 – 6.98 (m, 2H), 6.87 (d, J = 8.8 Hz, 2H), 6.74 – 6.66 (m, 2H), 6.40 – 6.32 (m, 4H), 6.27 (t, J = 7.3 Hz, 2H), 5.30 (d, J = 8.4 Hz, 2H), 2.65 (s, 3H), 2.51 (s, 3H). ^13^C NMR (151 MHz, Chloroform-d) δ 160.54, 156.38, 140.24, 140.02, 138.91, 138.27, 137.79, 137.74, 137.55, 136.04, 135.86, 134.62, 132.58, 132.18, 131.99, 129.48, 129.42, 129.35, 128.96, 128.13, 127.31, 127.29, 126.58, 126.51, 126.33, 126.04, 125.66, 124.67, 124.02, 121.29, 120.29, 119.93, 119.61, 113.97, 56.64, 15.87, 15.70.

**
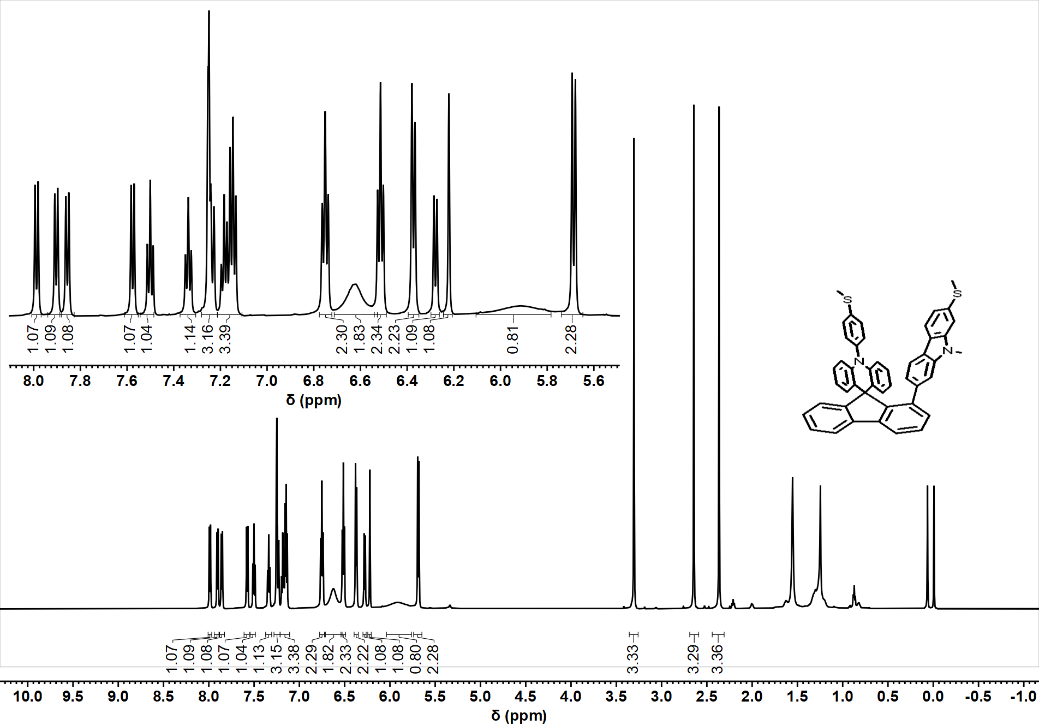
**

**
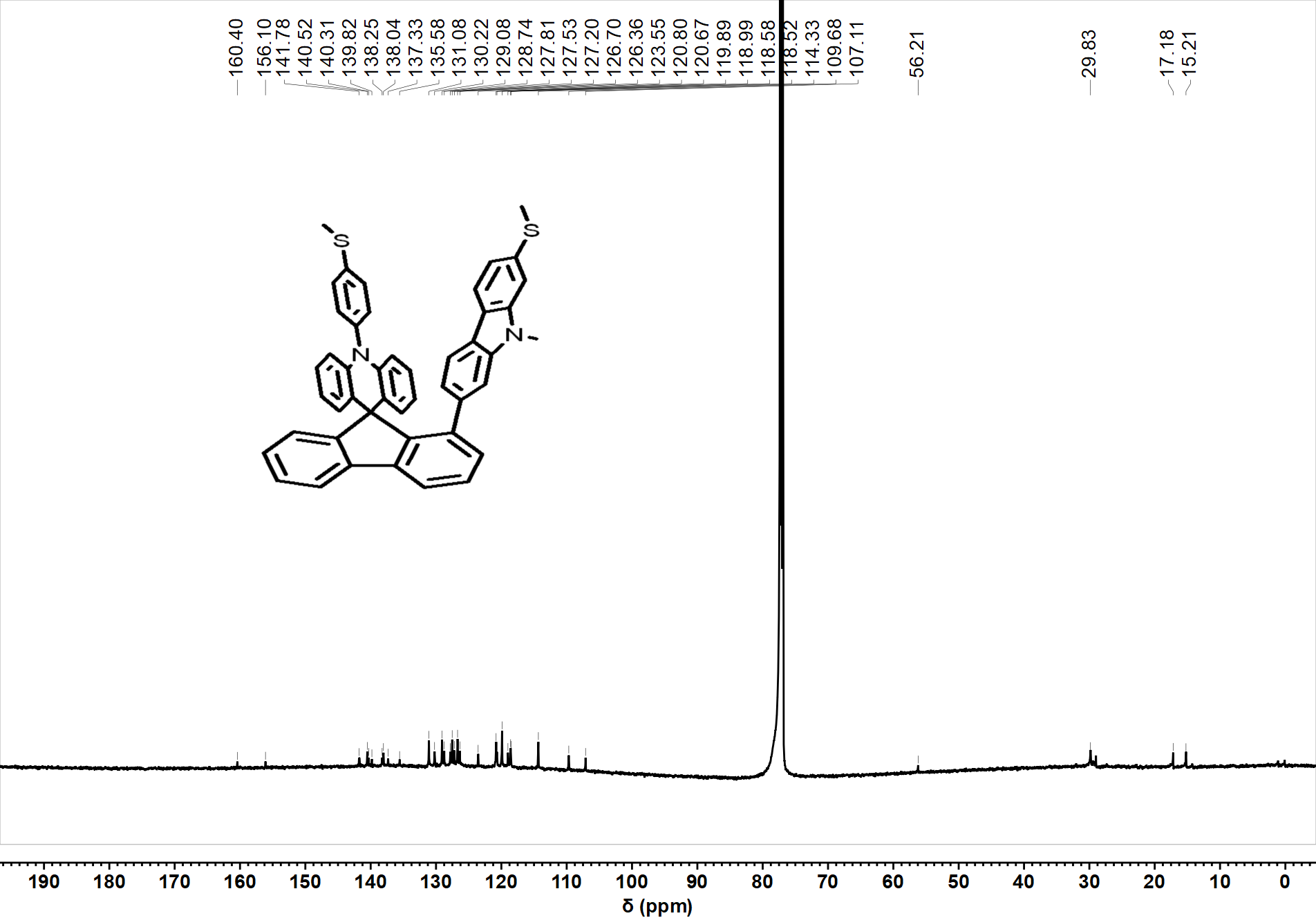
**

**Figure S7.** ^1^H NMR (top) and ^13^C NMR (bottom) spectra of TS3 in CDCl_3_ at room temperature. ^1^H NMR (600 MHz, Chloroform-d) δ 7.99 (d, J = 8.0 Hz, 1H), 7.90 (d, J = 7.6 Hz, 1H), 7.86 (d, J = 7.7 Hz, 1H), 7.58 (d, J = 7.8 Hz, 1H), 7.50 (t, J = 7.5 Hz, 1H), 7.34 (t, J = 7.4 Hz, 1H), 7.29 – 7.22 (m, 3H), 7.17 (dt, J = 22.8, 7.4 Hz, 3H), 6.75 (t, J = 7.7 Hz, 2H), 6.63 (s, 2H), 6.51 (t, J = 7.4 Hz, 2H), 6.37 (d, J = 7.7 Hz, 2H), 6.28 (d, J = 7.8 Hz, 1H), 6.22 (s, 1H), 5.91 (s, 1H), 5.69 (d, J = 8.4 Hz, 2H), 3.30 (s, 3H), 2.64 (s, 3H), 2.36 (s, 3H). ^13^C NMR (151 MHz, Chloroform-d) δ 160.41, 155.91, 141.79, 140.53, 140.31, 139.82, 138.26, 138.05, 137.33, 135.57, 134.98, 131.09, 130.22, 129.08, 128.75, 127.81, 127.54, 127.21, 126.70, 126.35, 123.55, 120.81, 120.68, 119.89, 119.00, 118.58, 114.34, 109.68, 107.12, 77.33, 77.12, 76.91, 56.22, 28.98, 17.19, 15.22.

**
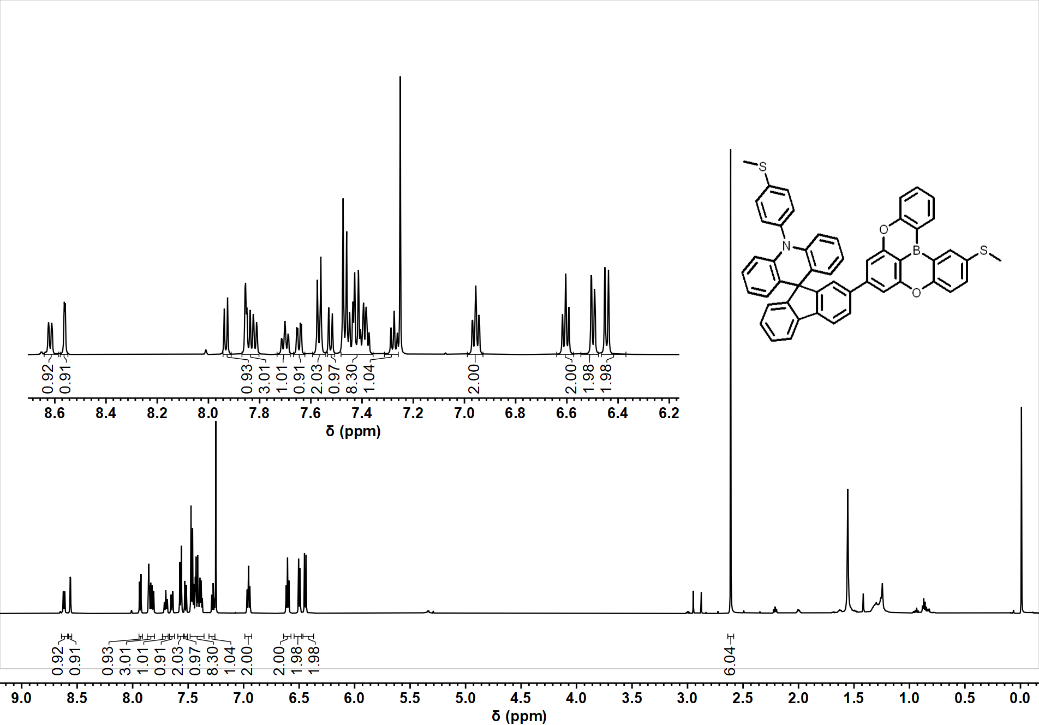
**

**
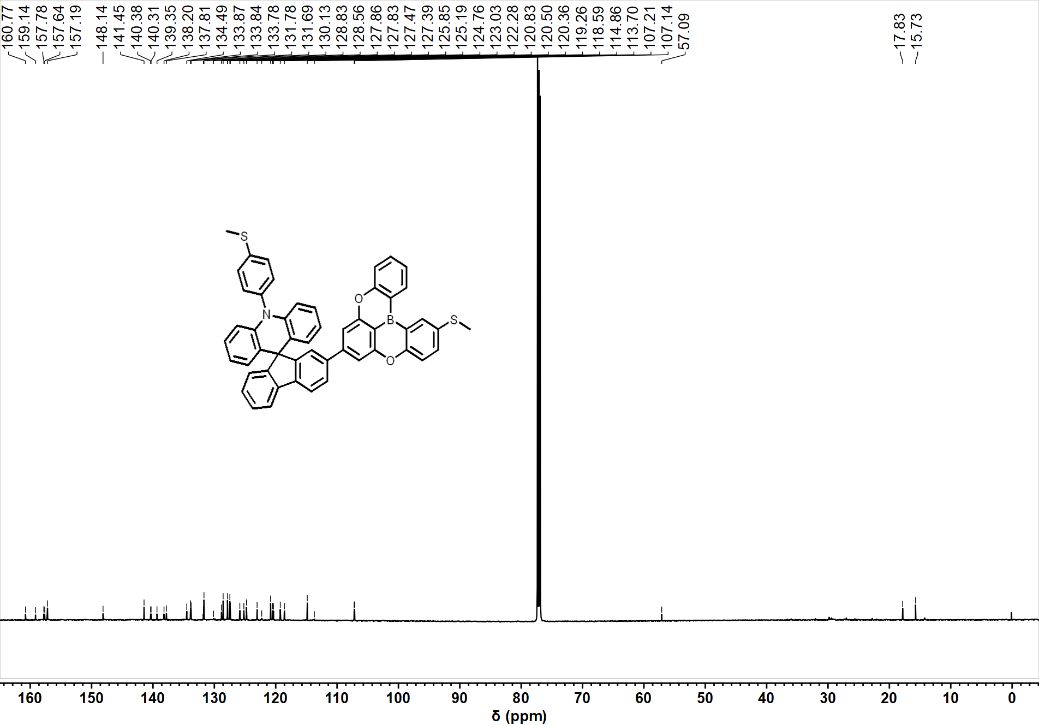
**

**Figure S8.** ^1^H NMR (top) and ^13^C NMR (bottom) spectra of TS4 in CDCl_3_ at room temperature.^1^H NMR (600 MHz, Chloroform-d) δ 8.62 (dd, J = 7.7, 1.7 Hz, 1H), 8.56 (d, J = 2.4 Hz, 1H), 7.93 (d, J = 7.8 Hz, 1H), 7.88 – 7.80 (m, 3H), 7.70 (ddd, J = 8.6, 7.0, 1.7 Hz, 1H), 7.65 (dd, J = 8.7, 2.3 Hz, 1H), 7.61 – 7.54 (m, 2H), 7.52 (d, J = 8.4 Hz, 1H), 7.48 – 7.37 (m, 8H), 7.30 – 7.26 (m, 1H), 6.96 (ddd, J = 8.5, 7.0, 1.6 Hz, 2H), 6.67 – 6.56 (m, 2H), 6.50 (dd, J = 7.9, 1.6 Hz, 2H), 6.46 – 6.40 (m, 2H), 2.61 (s, 6H). ^13^C NMR (151 MHz, Chloroform-d) δ 160.77, 159.14, 157.78, 157.64, 157.19, 148.14, 141.45, 140.38, 140.31, 139.35, 138.20, 137.81, 134.49, 133.87, 133.84, 133.78, 131.78, 131.69, 130.13, 128.83, 128.56, 127.86, 127.83, 127.47, 127.39, 125.85, 125.19, 124.76, 123.03, 122.28, 120.83, 120.50, 120.36, 119.26, 118.59, 114.86, 113.70, 107.21, 107.14, 57.09, 17.83, 15.73.

**
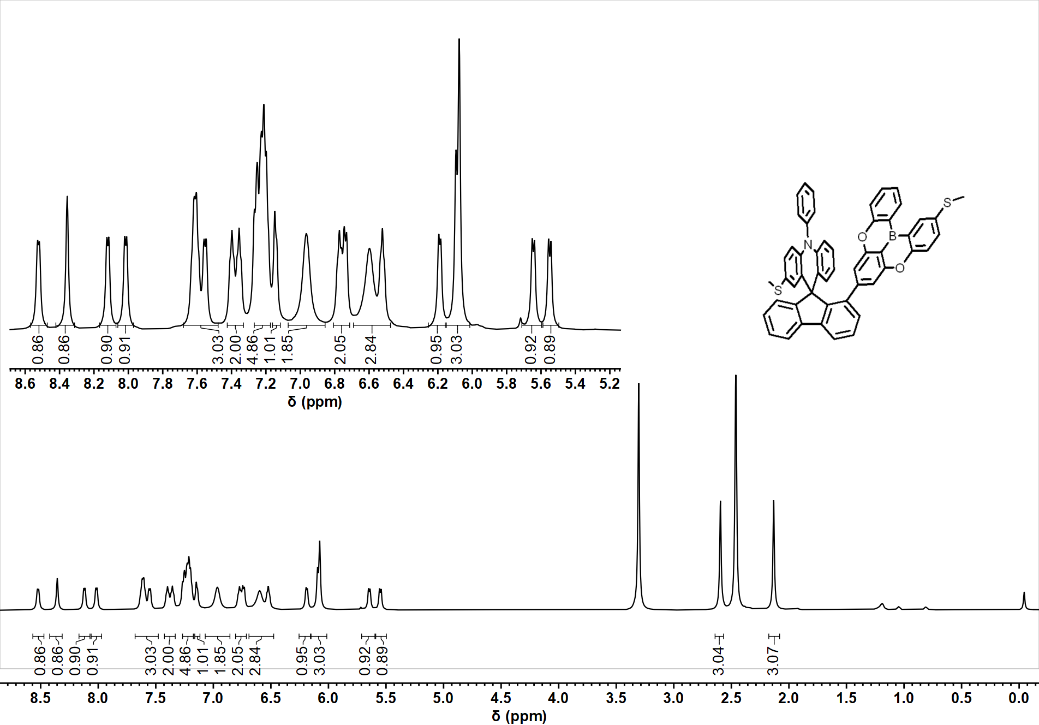
**

**
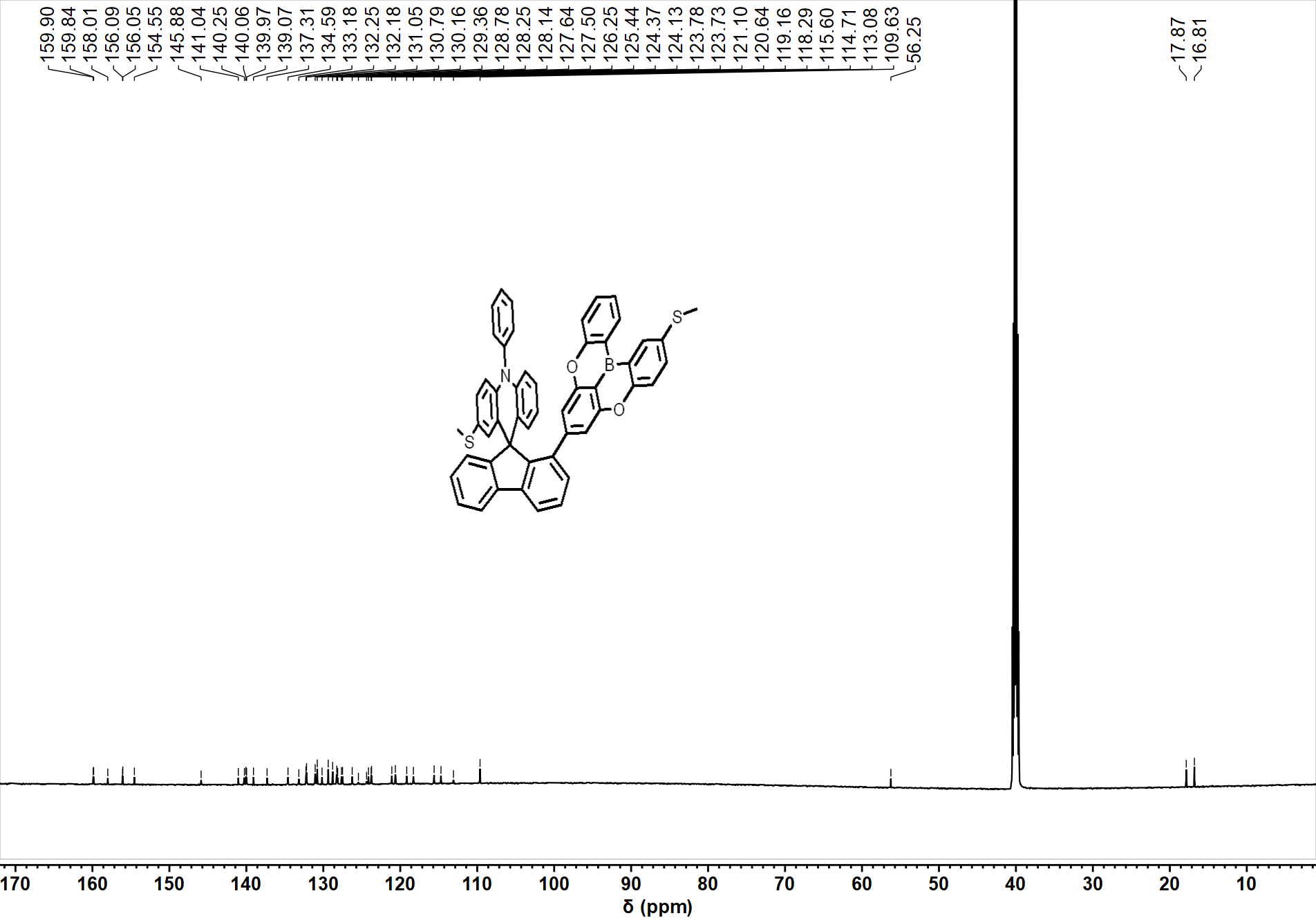
**

**Figure S9.** ^1^H NMR (top) and ^13^C NMR (bottom) spectra of TS5 in DMSO at room temperature. ^1^H NMR (600 MHz, DMSO-d6) δ 8.52 (d, J = 7.6 Hz, 1H), 8.36 (s, 1H), 8.12 (d, J = 7.7 Hz, 1H), 8.01 (d, J = 7.8 Hz, 1H), 7.69 – 7.50 (m, 3H), 7.38 (dt, J = 25.5, 7.4 Hz, 2H), 7.30 – 7.19 (m, 5H), 7.14 (d, J = 7.5 Hz, 1H), 6.96 (s, 2H), 6.87 – 6.71 (m, 2H), 6.63 – 6.48 (m, 3H), 6.19 (d, J = 7.8 Hz, 1H), 6.09 (d, J = 11.6 Hz, 3H), 5.64 (d, J = 8.5 Hz, 1H), 5.55 (d, J = 8.8 Hz, 1H), 2.59 (s, 3H), 2.13 (s, 3H). ^13^C NMR (151 MHz, DMSO-d6) δ 159.90, 159.84, 158.01, 156.09, 156.05, 154.55, 145.88, 141.04, 140.25, 140.06, 139.97, 139.07, 137.31, 134.59, 133.18, 132.25, 132.18, 131.05, 130.79, 130.16, 129.36, 128.78, 128.25, 128.14, 127.64, 127.50, 126.25, 125.44, 124.37, 124.13, 123.78, 123.73, 121.10, 120.64, 119.16, 118.29, 115.60, 114.71, 113.08, 109.63, 56.25, 17.87, 16.81.

**
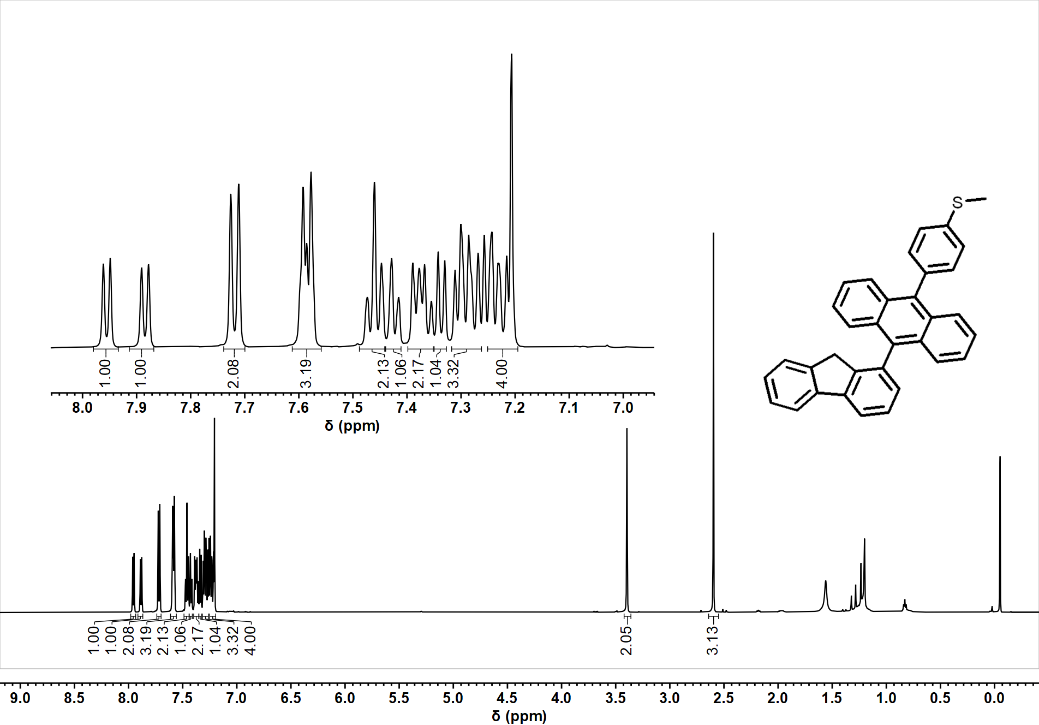
**

**
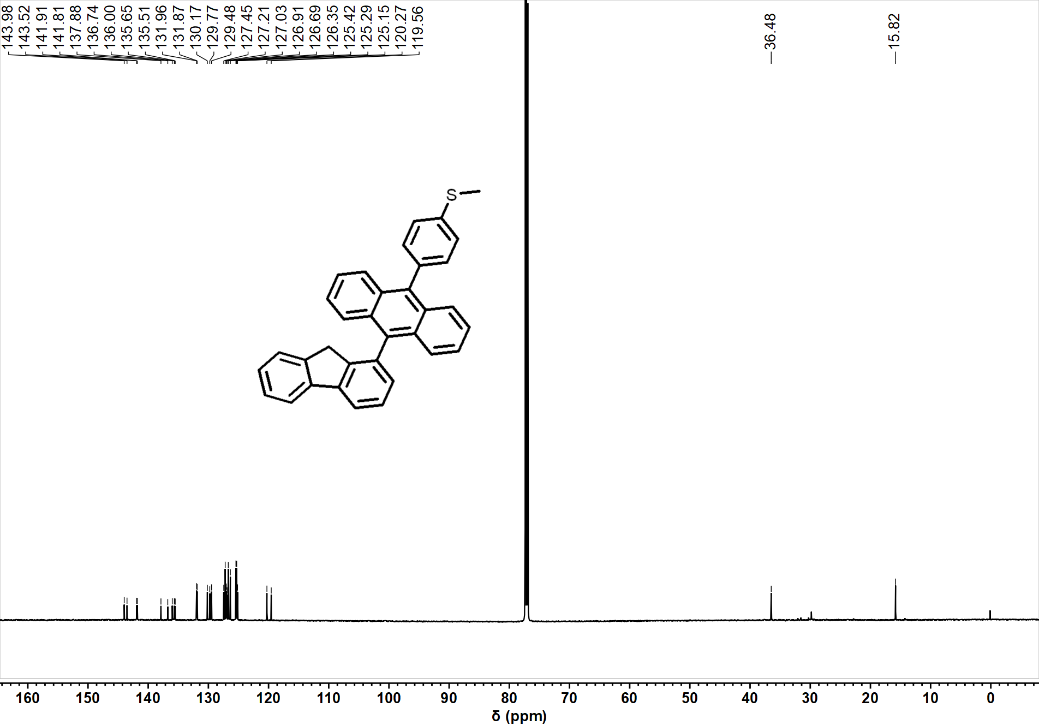
**

**Figure S10.** ^1^H NMR (top) and ^13^C NMR (bottom) spectra of wu-EN-S in CDCl_3_ at room temperature. ^1^H NMR (600 MHz, Chloroform-d) δ 8.00 (d, J = 7.6 Hz, 1H), 7.93 (d, J = 7.7 Hz, 1H), 7.76 (d, J = 8.8 Hz, 2H), 7.62 (dd, J = 8.1, 3.4 Hz, 3H), 7.53 – 7.48 (m, 2H), 7.46 (dd, J = 8.7, 1.5 Hz, 1H), 7.41 (dd, J = 13.4, 7.4 Hz, 2H), 7.38 (d, J = 7.4 Hz, 1H), 7.36 – 7.29 (m, 4H), 7.29 – 7.25 (m, 2H), 3.44 (s, 2H), 2.64 (d, J = 1.2 Hz, 3H). ^13^C NMR (151 MHz, Chloroform-d) δ 143.98, 143.52, 141.91, 141.81, 137.88, 136.74, 136.00, 135.65, 135.51, 131.96, 131.87, 130.17, 129.77, 129.48, 127.45, 127.21, 127.03, 126.91, 126.69, 126.35, 125.42, 125.29, 125.15, 120.27, 119.56, 36.48, 15.82.

**
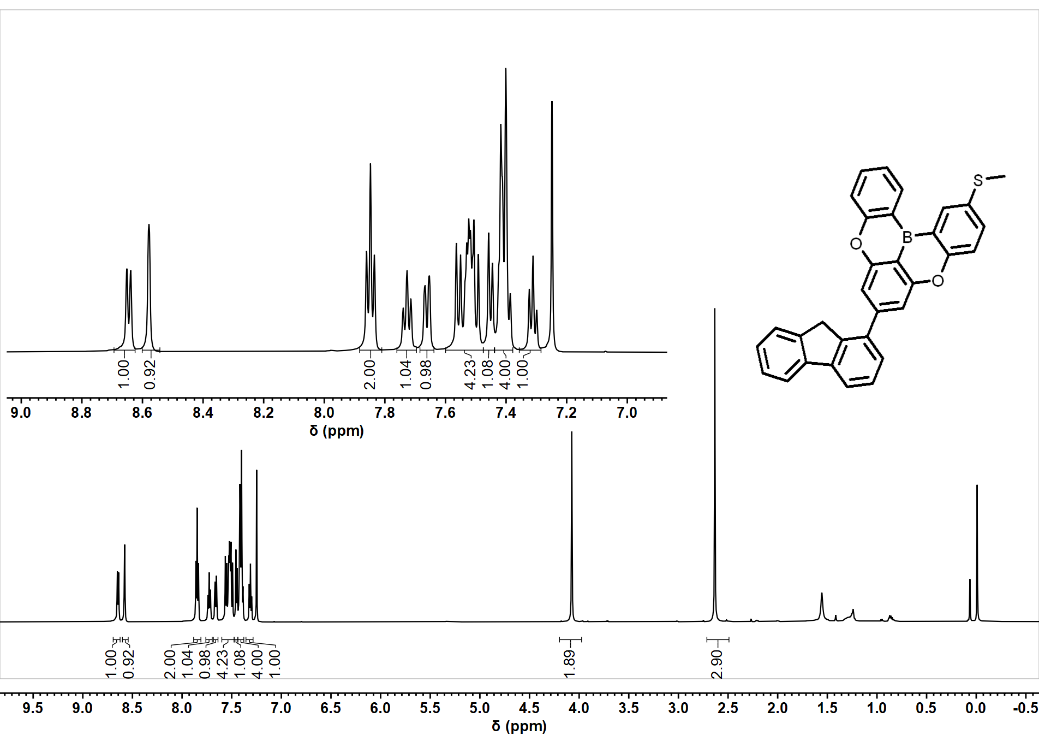
**

**
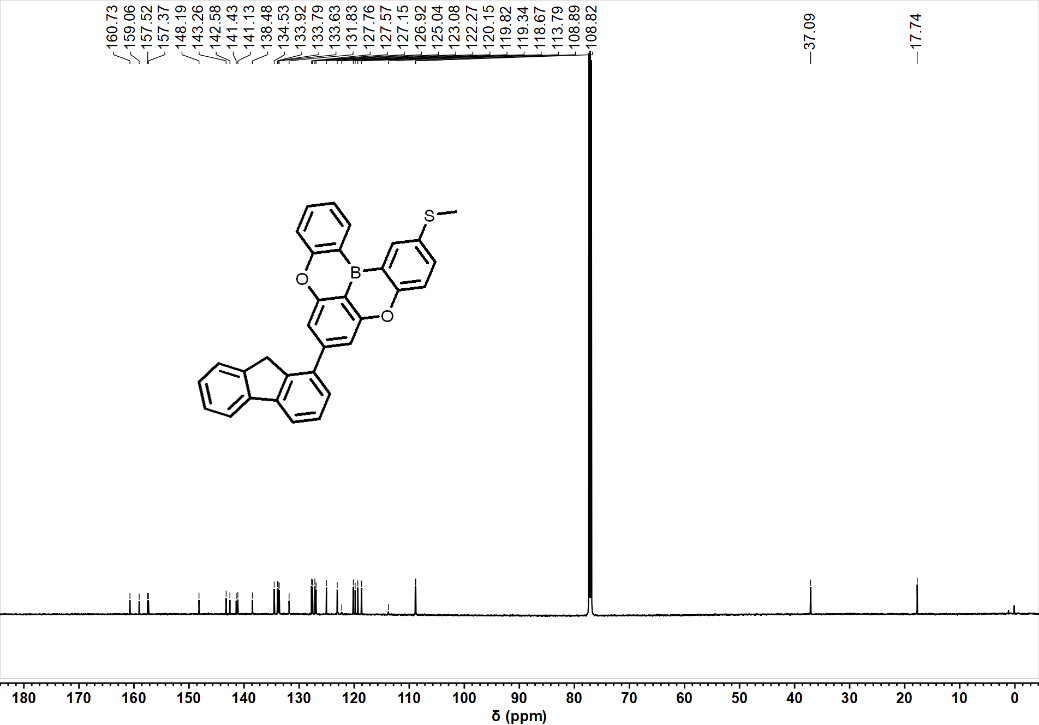
**

**Figure S11.** ^1^H NMR (top) and ^13^C NMR (bottom) spectra of wu-BO-S in CDCl_3_ at room temperature. ^1^H NMR (600 MHz, Chloroform-d) δ 8.65 (d, J = 7.7 Hz, 1H), 8.58 (d, J = 2.4 Hz, 1H), 7.85 (t, J = 7.8 Hz, 2H), 7.73 (td, J = 7.8, 7.0, 1.6 Hz, 1H), 7.69 – 7.63 (m, 1H), 7.56 (d, J = 8.3 Hz, 1H), 7.54 – 7.48 (m, 3H), 7.45 (d, J = 7.5 Hz, 1H), 7.43 – 7.37 (m, 4H), 7.31 (t, J = 7.4 Hz, 1H), 4.07 (s, 2H), 2.63 (s, 3H). ^13^C NMR (151 MHz, Chloroform-d) δ 160.73, 159.06, 157.52, 157.37, 148.19, 143.26, 142.58, 141.43, 141.13, 138.48, 134.53, 133.92, 133.79, 133.63, 131.83, 127.76, 127.57, 127.15, 126.92, 125.04, 123.08, 122.27, 120.15, 119.82, 119.34, 118.67, 113.79, 108.89, 108.82, 37.09, 17.74.

**
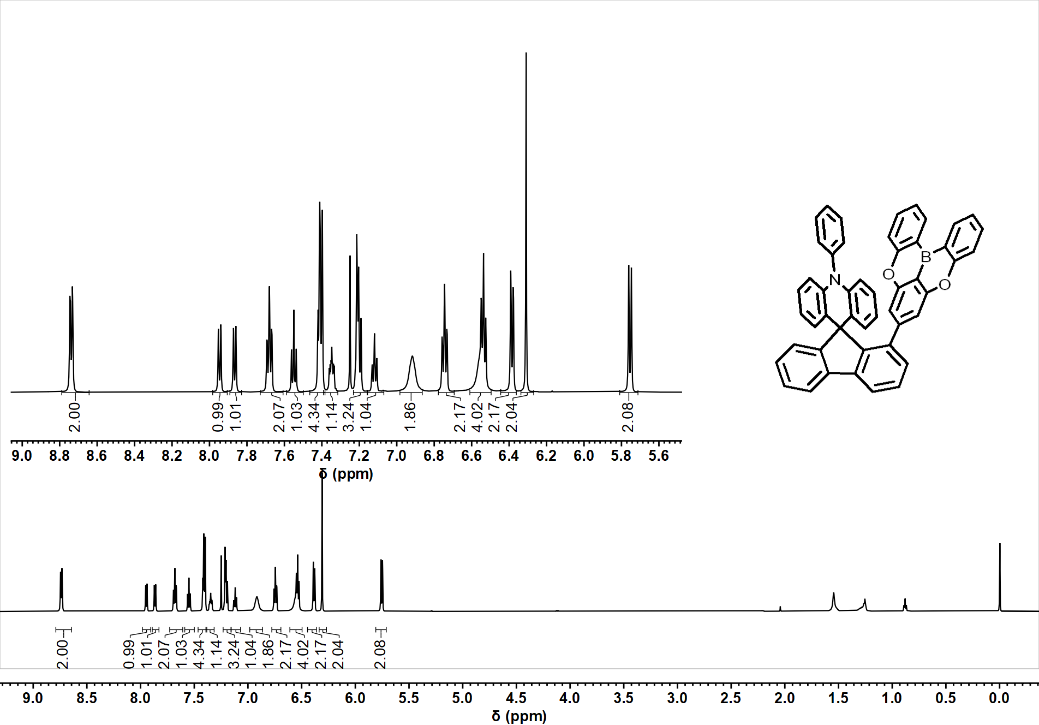
**

**
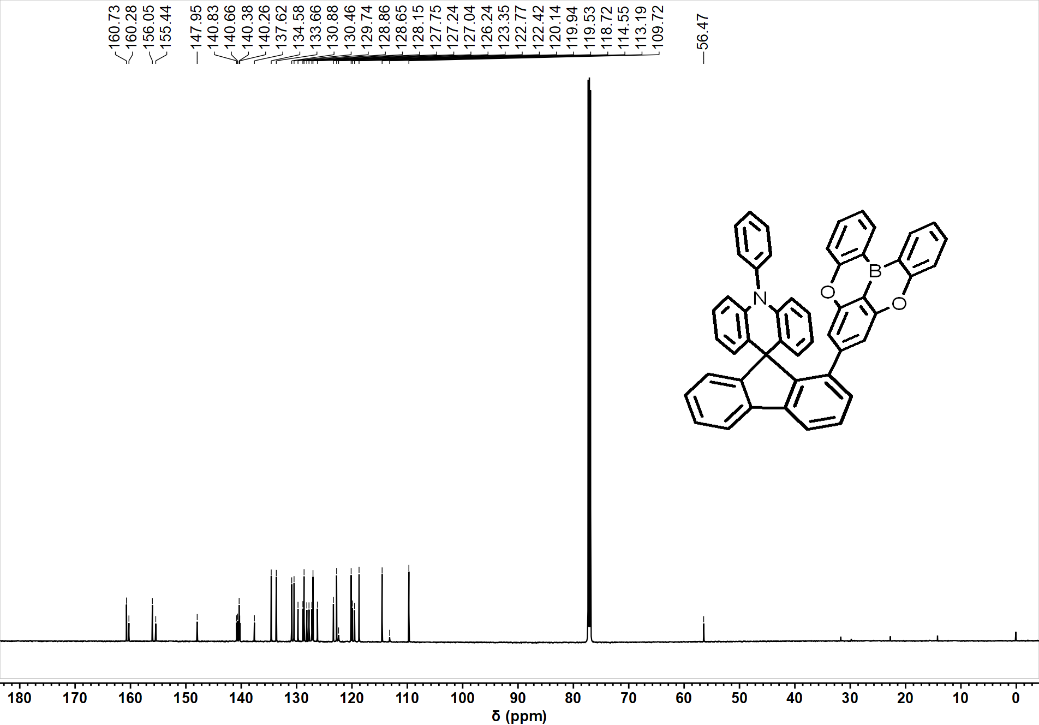
**

**Figure S12.** ^1^H NMR (top) and ^13^C NMR (bottom) spectra of REF in CDCl_3_ at room temperature. ^1^H NMR (600 MHz, Chloroform-d) δ 8.74 (dd, J = 7.9, 1.8 Hz, 1H), 7.95 (dd, J = 7.7, 1.1 Hz, 1H), 7.87 (d, J = 7.7 Hz, 1H), 7.68 (ddd, J = 8.5, 7.0, 1.7 Hz, 1H), 7.55 (t, J = 7.6 Hz, 1H), 7.45 – 7.38 (m, 2H), 7.35 (ddd, J = 8.0, 5.7, 2.7 Hz, 1H), 7.24 – 7.16 (m, 2H), 7.12 (td, J = 7.3, 6.3, 1.3 Hz, 1H), 6.92 (s, 1H), 6.74 (ddd, J = 8.5, 7.0, 1.6 Hz, 1H), 6.54 (td, J = 7.6, 3.7 Hz, 2H), 6.38 (dd, J = 7.8, 1.6 Hz, 1H), 6.31 (s, 1H), 5.75 (dd, J = 8.4, 1.2 Hz, 1H). ^13^C NMR (151 MHz, Chloroform-d) δ 160.73, 160.28, 156.05, 155.44, 147.95, 140.83, 140.66, 140.38, 140.26, 137.62, 134.58, 133.66, 130.88, 130.46, 129.74, 128.86, 128.65, 128.15, 127.75, 127.24, 127.04, 126.24, 123.35, 122.77, 122.42, 120.14, 119.94, 119.53, 118.72, 114.55, 113.19, 109.72, 77.33, 77.12, 76.91, 56.47.


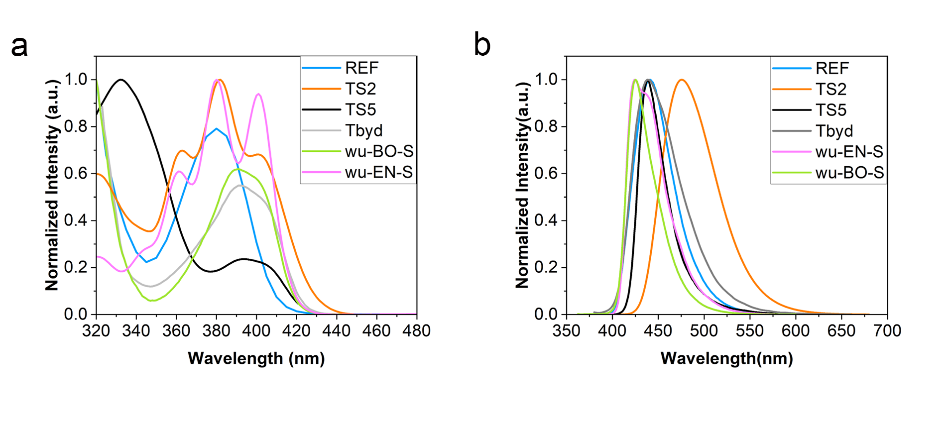


**Figure S13.** Absorption spectra of REF, TS2, TS5, Tbyd, wu-EN-S and wu-BO-S. a) Normalized absorption (room temperature, 1×10^-5^ M in mesitylene) of REF, TS2, TS5, Tbyd, wu-EN-S and wu-BO-S. b) Normalized fluorescence (room temperature, 1×10^-5^ M in mesitylene) of REF, TS2, TS5, Tbyd, wu-EN-S and wu-BO-S.


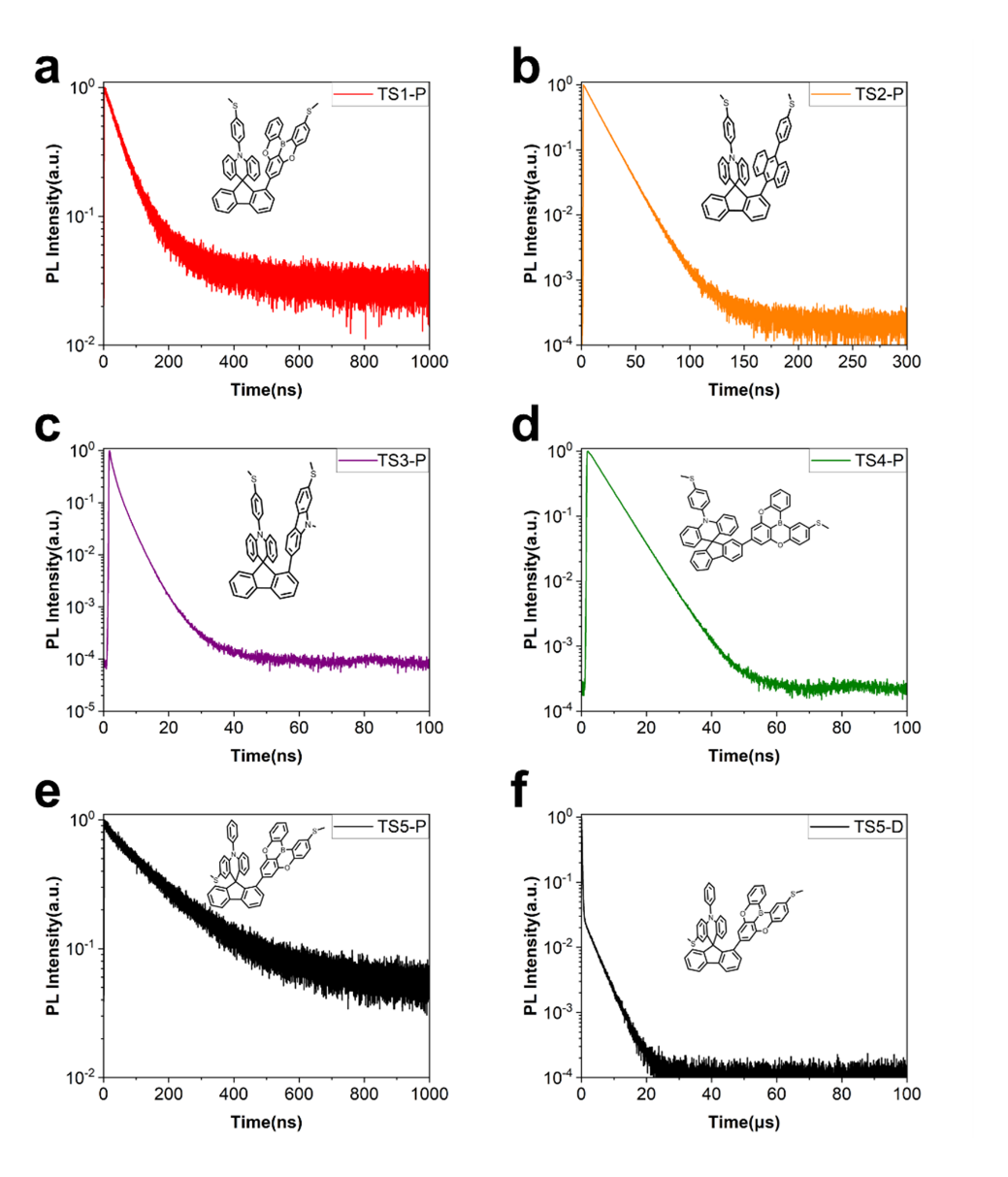


**Figure S14.** Transient photoluminescence (PL) decay characteristics of TS1, TS2, TS3, TS4 and TS5. a-e), Prompt components of transient PL spectra of TS1, TS2, TS3, TS4 and TS5. f) Delayed component of transient PL spectra of TS5.


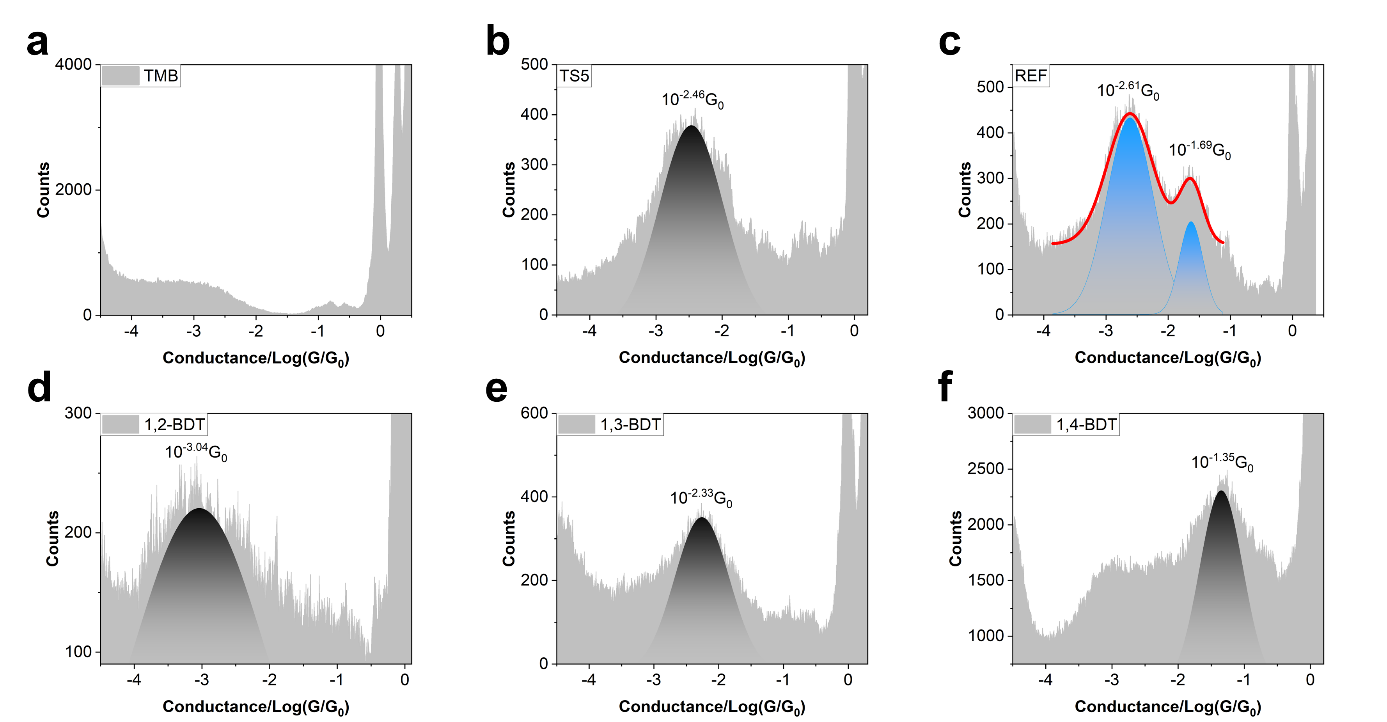


**Figure S15.** Conductance measurements of the probe and control molecules. a) pure mesitylene solvent (TMB). b) TS5. c) REF. d) 1,2-biphenyldithiol (1,2-BDT). e) 1,3-biphenyldithiol (1,3-BDT). f) 1,4-biphenyldithiol (1,4-BDT).


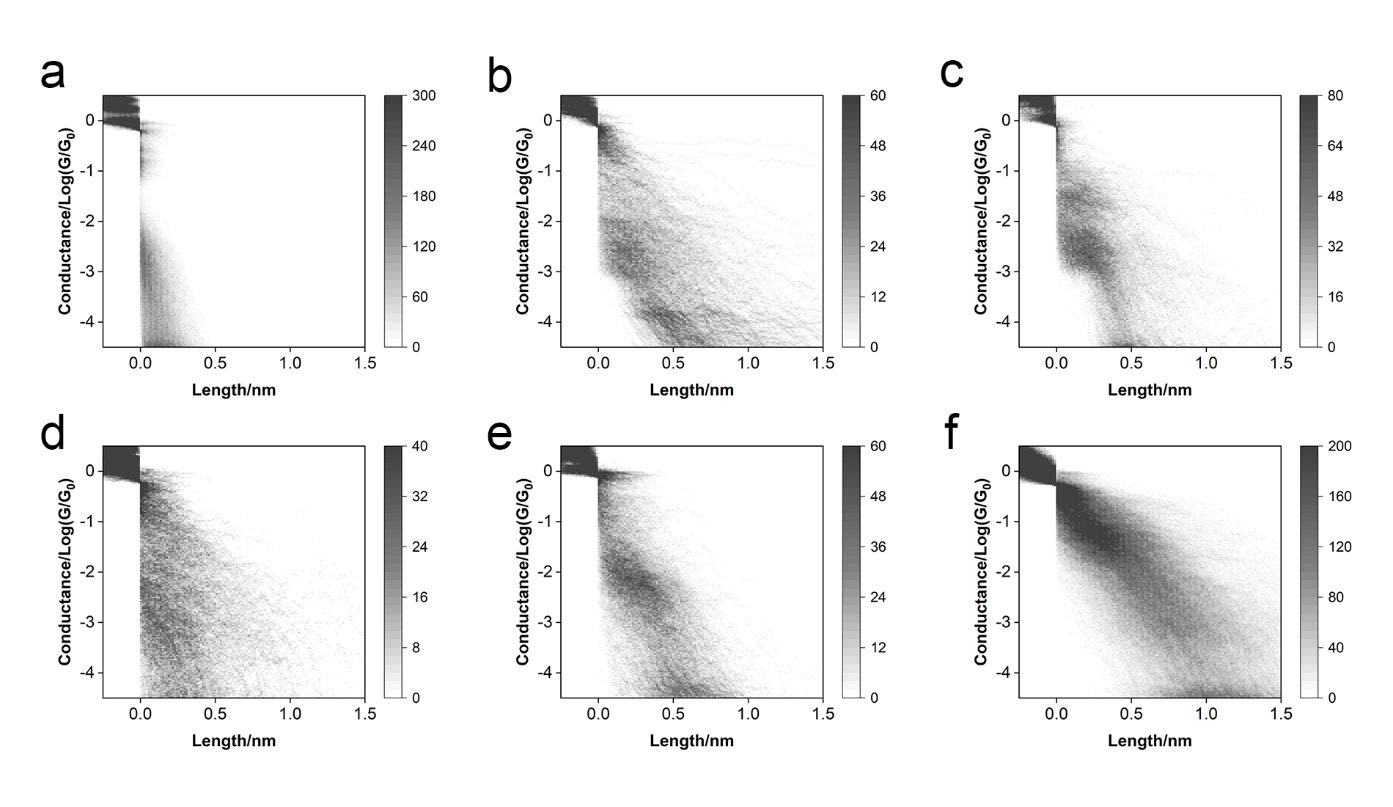


**Figure S16.** Two-dimensional (2D) conductance histograms of conductance versus distance of control molecules. a) mesitylene (TMB). b) TS5. c) REF. d) 1,2-biphenyldithiol (1,2-BDT). e) 1,3-biphenyldithiol (1,3-BDT). f) 1,4-biphenyldithiol (1,4-BDT).


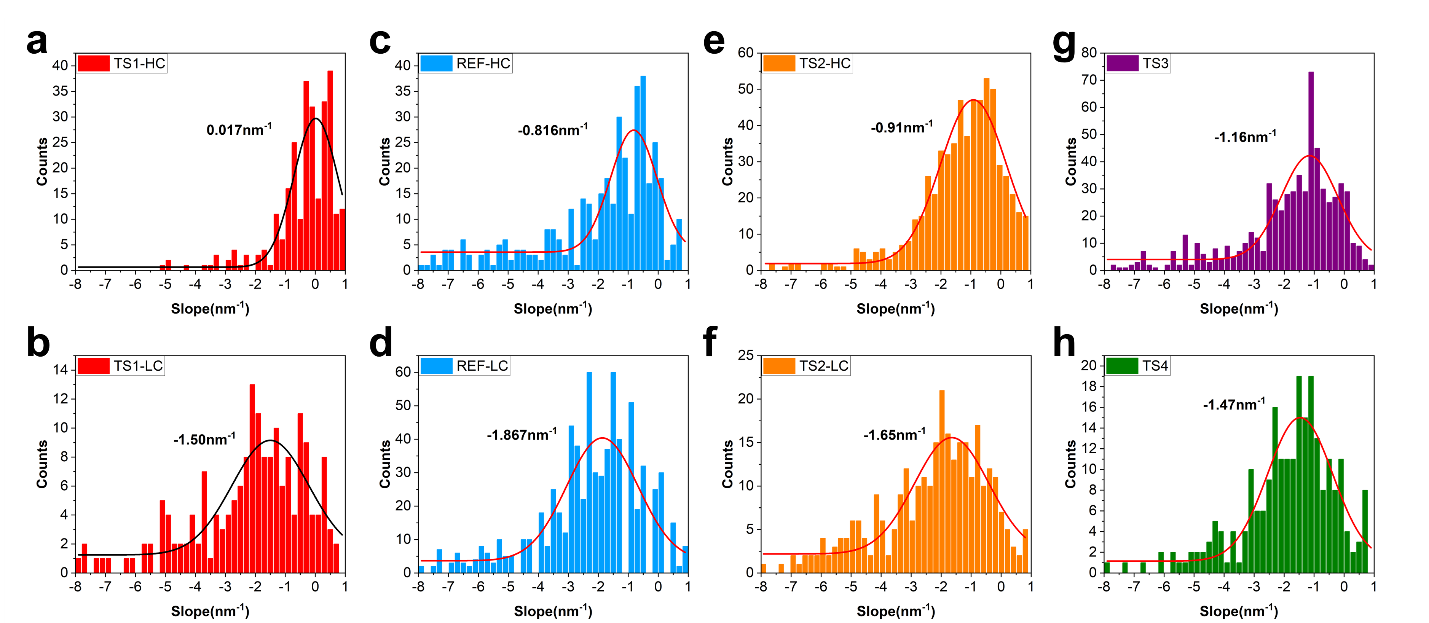


**Figure S17.** Distribution of plateau slopes for TS1, TS2, TS3, TS4 and REF. a) TS1-HC. b) TS1-LC. c) REF-HC. d) REF-LC. e) TS2-HC. f) TS2-LC. g) TS3. h) TS4.


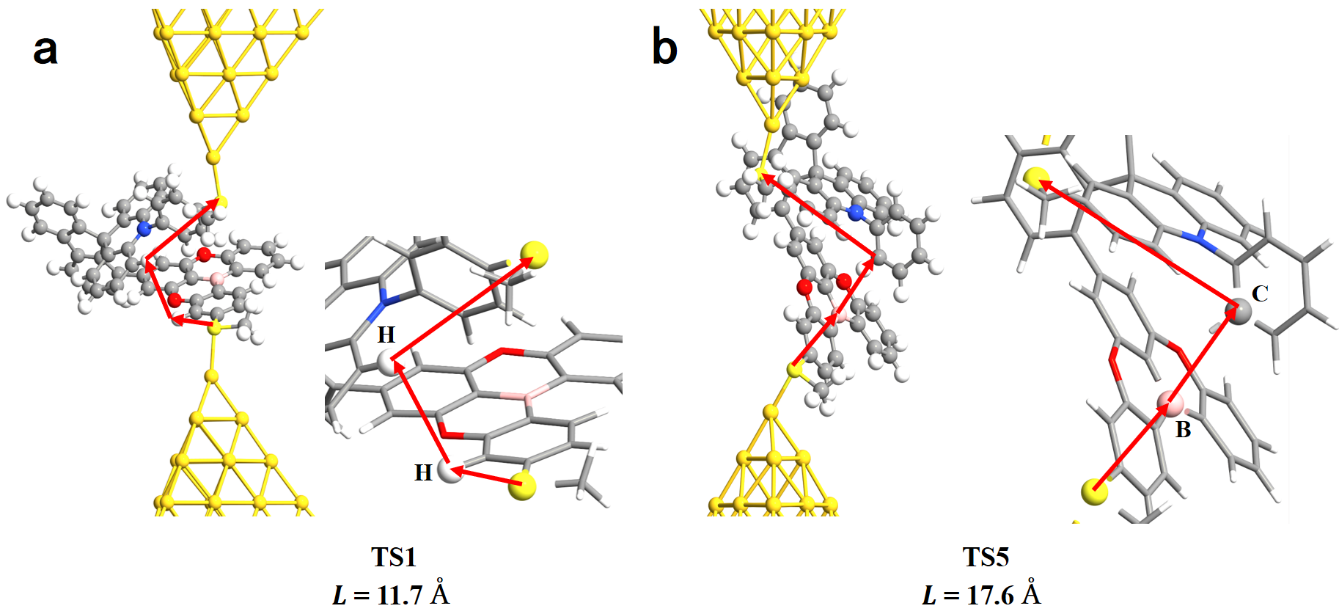


**Figure S18.** Schematic of the calculated dominant through-space transmission pathways linking the electrode-bonded S atoms via the specific donor-acceptor atom pair that provides the highest local bond contributions to the transmission coefficients. The length of the pathway is denoted as *L*.


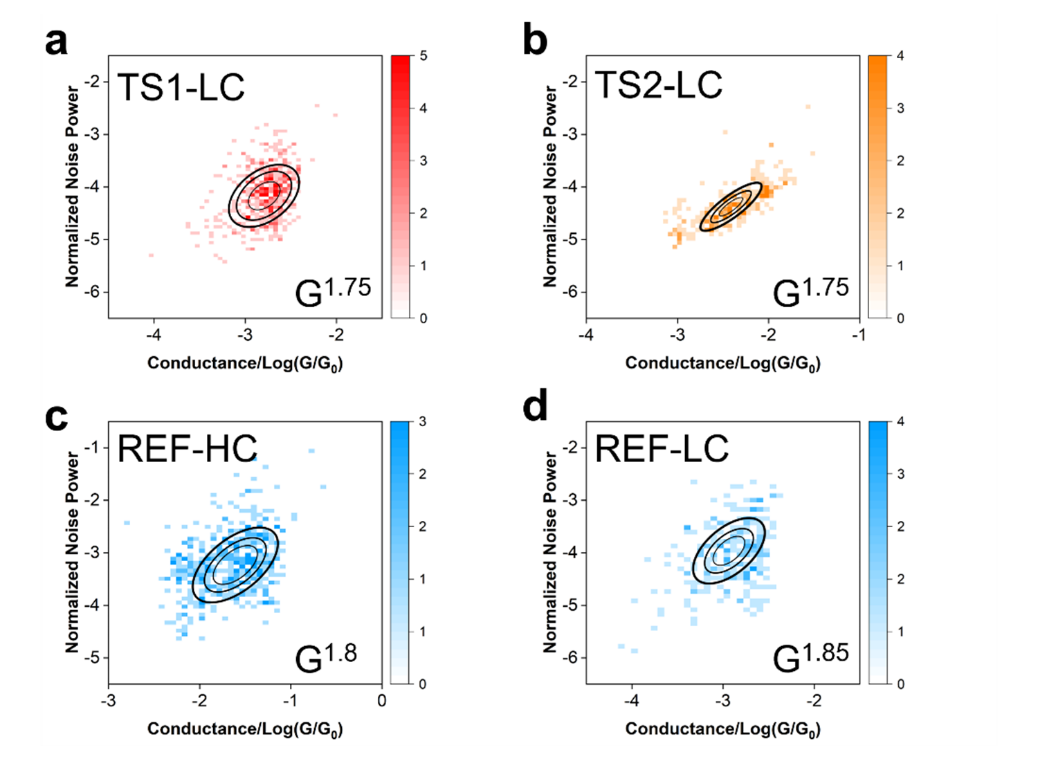


**Figure S19.** Analysis of flicker noise power of TS1, TS2 and REF. a) Low-conductance state of TS1 (TS1-LC). b) Low-conductance state of TS2 (TS2-LC). c) High-conductance state of REF. d) Low-conductance state of REF.


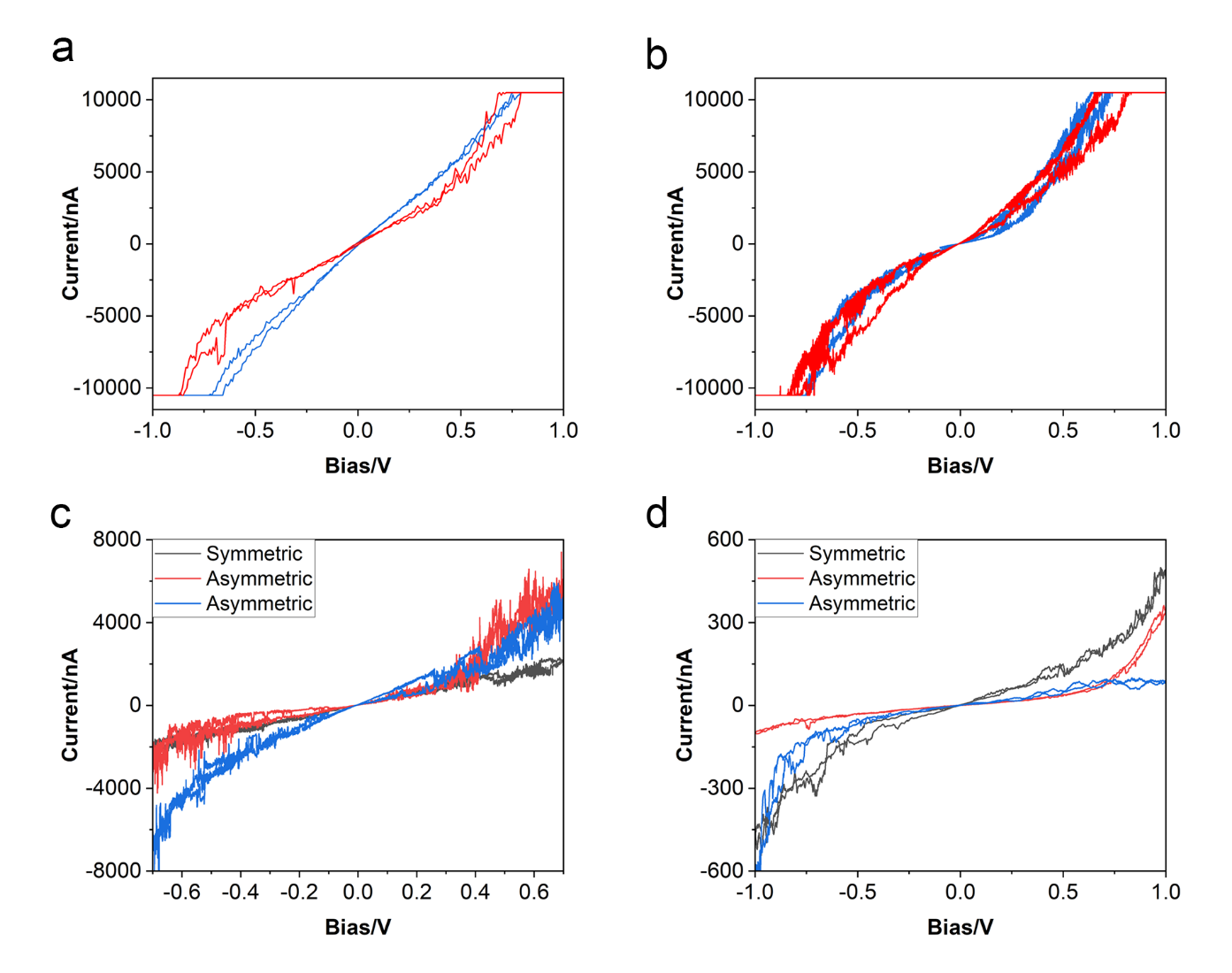


**Figure S20.** Individual current-voltage curves exceeding the upper detection limit for TS1 (HC) (a) and TS2 (HC) (b). Individual current-voltage curves with different symmetries of TS2 (HC) (c) and TS3 (d).


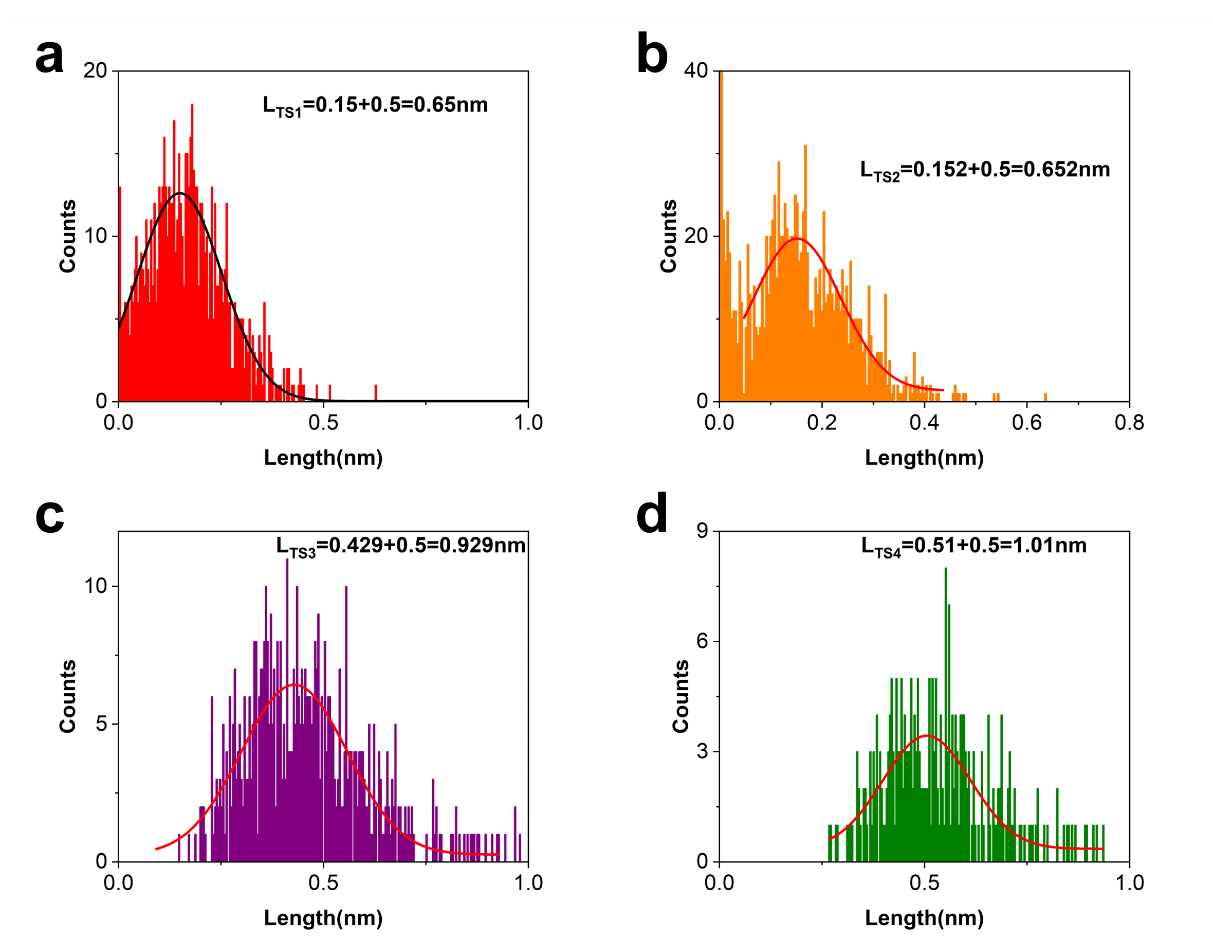


**Figure S21.** Distribution of total tip displacement for (a) TS1, (b) TS2, (c) TS3 and (d) TS4 junction upon breakdown.


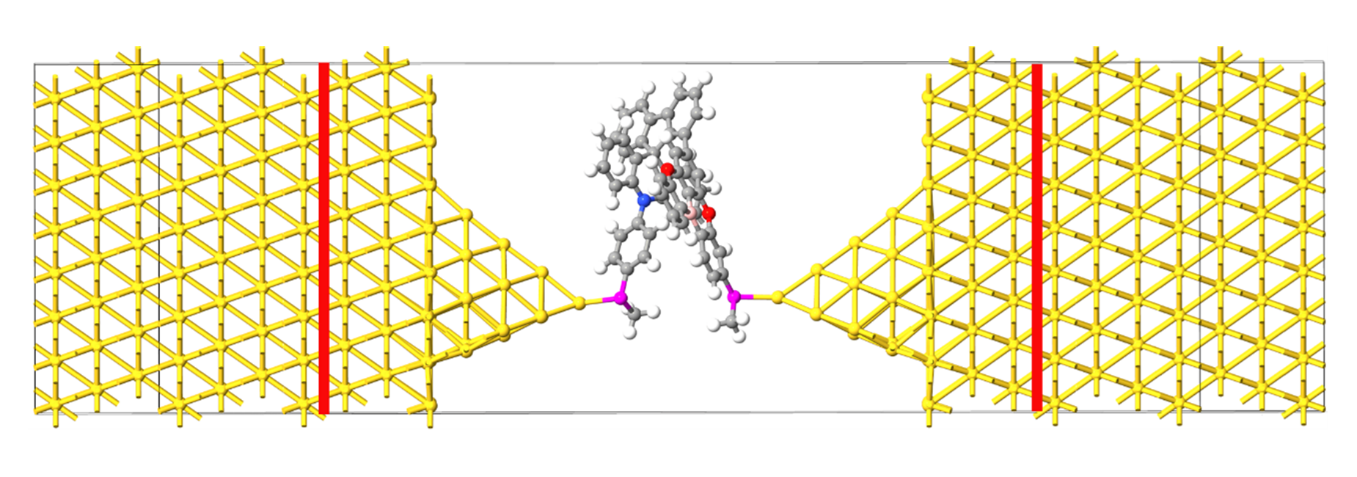


**Figure S22.** Configuration of the molecular junction model for electron transport calculations. The region between the two red vertical lines corresponds to the optimized structure. The Au, C, N, O, B, S and H atoms are depicted in yellow, gray, blue, red, pink, fuchsia, and white, respectively.


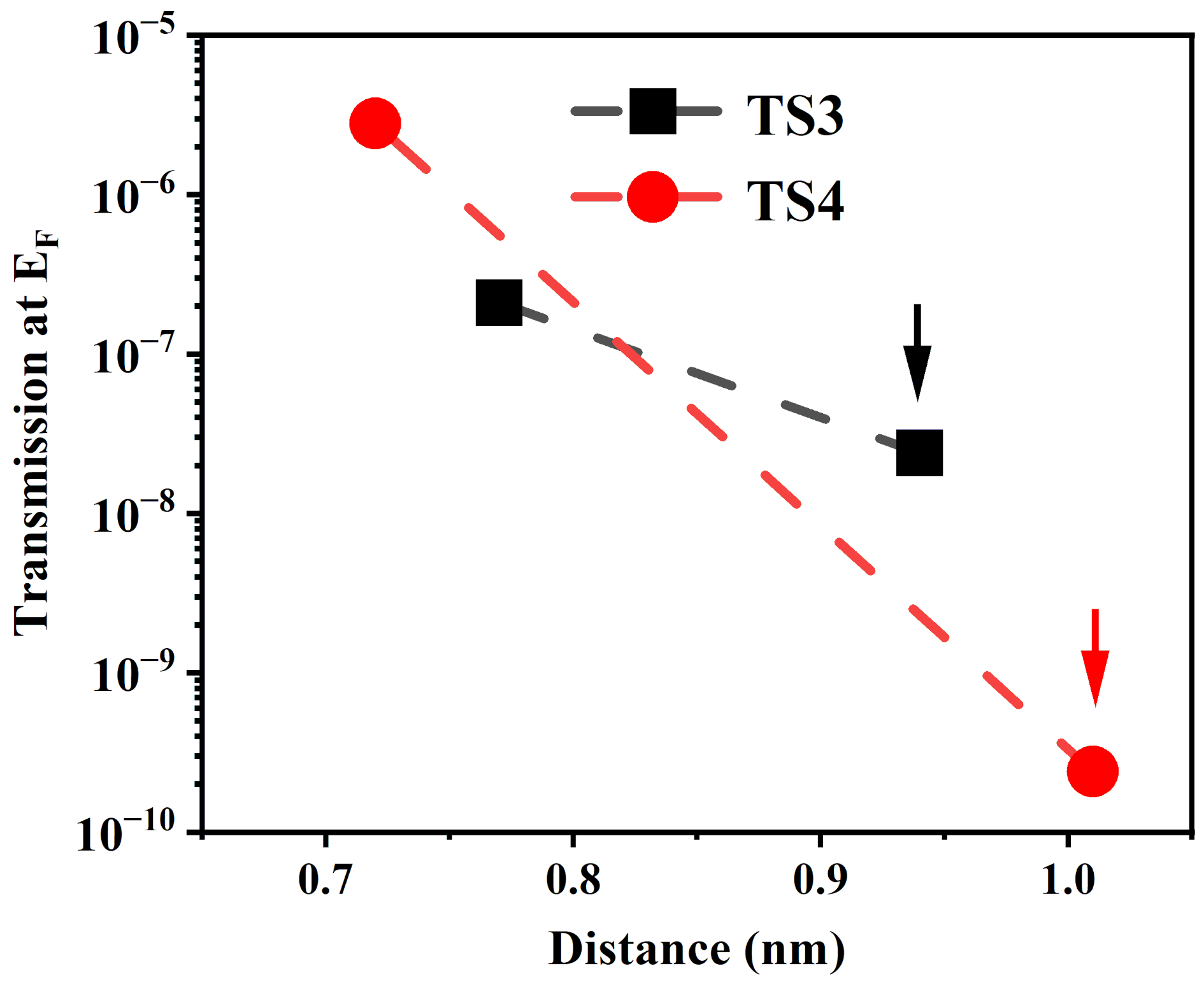


**Figure S23.** Calculated transmission coefficients of TS3 and TS4 at their respective Fermi levels for two different electrode distances. Arrows indicate the experimentally measured rupture distances. Dashed lines are provided as visual guides.

**Supporting Table**

**Table S1.** Summary of spectral characterization of all target molecules.

| Molecules | λ^a)^_abs_(nm) | λ_PL_(nm) | FWHM^b)^(nm) | τ_p_ (ns) | τ_d_ (μs) | PLQE (%) |
| --- | --- | --- | --- | --- | --- | --- |
| TS1 | 453 | 451 | 60 | 67 | 15.6 | 92 |
| TS2 | 457 | 475 | 68 | 14 | - | 18 |
| TS3 | 407 | 381 | 42 | 1.8 | - | 11 |
| TS4 | 438 | 438 | 46 | 5.4 | - | 37 |
| TS5 | 435 | 438 | 34 | 159.6 | 3.51 | 95 |
| REF | 430 | 441 | 50 | - | - | - |
| wu-EN-S | 426 | 424 | 45 | - | - | - |
| wu-BO-S | 440 | 425 | 35 | - | - | - |
| Tbyd | 436 | 438 | 58 | - | - | - |

a) Cut-off absorption wavelength; b) Full width at half-maximum.

**Table S2.** Results of estimated spring constants of TS1, TS2, TS3.

| Molecules | TS1 | TS2 | TS3 |
| --- | --- | --- | --- |
| k_eff_ (N/m) | 26.03 | 12.52 | 2.79 |
| k_b_ (N/m) | $\text{≤2.79}$ | $\text{≤2.79}$ | $\text{≤2.79}$ |
| k_i_ (N/m) | $\text{≥23.24}$ | $\text{≥9.73}$ | $\text{≥0}$ |
